# Cytokines as therapeutic targets for cardio- and cerebrovascular diseases

**DOI:** 10.1007/s00395-021-00863-x

**Published:** 2021-03-26

**Authors:** Luca Liberale, Stefano Ministrini, Federico Carbone, Giovanni G. Camici, Fabrizio Montecucco

**Affiliations:** 1 Center for Molecular Cardiology, University of Zürich, Wagistrasse 12, 8952 Schlieren, Switzerland; 2 First Clinic of Internal Medicine, Department of Internal Medicine, University of Genoa, Genoa, Italy; 3 Internal Medicine, Angiology and Atherosclerosis, Department of Medicine and Surgery, University of Perugia, Perugia, Italy; 4 IRCCS Ospedale Policlinico San Martino Genoa, Italian Cardiovascular Network, Genoa, Italy; 5 Department of Cardiology, University Heart Center, University Hospital Zurich, Zurich, Switzerland; 6 Department of Research and Education, University Hospital Zurich, Zurich, Switzerland; 7 First Clinic of Internal Medicine, Department of Internal Medicine and Centre of Excellence for Biomedical Research (CEBR), University of Genoa, Genoa, Italy

**Keywords:** Cytokines, Interleukins, IL-1, IL-6, Cardiovascular disease, Cerebrovascular disease

## Abstract

Despite major advances in prevention and treatment, cardiac and cerebral atherothrombotic complications still account for substantial morbidity and mortality worldwide. In this context, inflammation is involved in the chronic process leading atherosclerotic plaque formation and its complications, as well as in the maladaptive response to acute ischemic events. For this reason, modulation of inflammation is nowadays seen as a promising therapeutic strategy to counteract the burden of cardio- and cerebrovascular disease. Being produced and recognized by both inflammatory and vascular cells, the complex network of cytokines holds key functions in the crosstalk of these two systems and orchestrates the progression of atherothrombosis. By binding to membrane receptors, these soluble mediators trigger specific intracellular signaling pathways eventually leading to the activation of transcription factors and a deep modulation of cell function. Both stimulatory and inhibitory cytokines have been described and progressively reported as markers of disease or interesting therapeutic targets in the cardiovascular field. Nevertheless, cytokine inhibition is burdened by harmful side effects that will most likely prevent its chronic use in favor of acute administrations in well-selected subjects at high risk. Here, we summarize the current state of knowledge regarding the modulatory role of cytokines on atherosclerosis, myocardial infarction, and stroke. Then, we discuss evidence from clinical trials specifically targeting cytokines and the potential implication of these advances into daily clinical practice.

## Introduction

Cardiovascular (CV) pharmacology underwent major advances in the last decades allowing for a more effective prevention of cardiovascular and cerebrovascular events. Yet, acute thrombotic complications of atherosclerosis such as ischemic stroke and myocardial infarction remain global leading causes of disability and mortality. With more than 32.4 million myocardial infarctions and strokes worldwide every year, the potential for intervention is high and requires greater efforts [34]. Lipids and inflammation have long been considered key drivers of plaque growth and rupture [15, 146]. While statin-based lipid control effectively reduced CV burden in high-risk patients [140], the recent introduction of Proprotein Convertase Subtilisin/Kexin Type (PCSK) 9 inhibitors definitively addressed the CV risk related to circulating cholesterol particles [144]. Yet, despite optimal low-density lipoprotein cholesterol (LDL) reduction, some patients still present a residual CV risk and may benefit from additional drugs targeting other pathways involved in atherogenesis [133].

The inflammatory theory of atherosclerosis was first proposed based on histological observation in the early 80s [195], while only recently the Canakinumab Antiinflammatory Thrombosis Outcome Study (CANTOS) demonstrated the efficacy of specific anti-inflammatory intervention in the setting of secondary CV prevention and confirmed inflammatory mediators to be crucial participants and effective targets in atherogenesis [192]. Cytokines are small soluble proteins with major autocrine, paracrine, and endocrine immunomodulatory functions. Monoclonal antibodies inhibiting cytokines (also referred to as biologic therapy or biologicals) and specifically anti-tumor necrosis factor (TNF)-α have first proved efficacy in the early 90s in the treatment of patients with methotrexate-resistant rheumatoid arthritis [65]. Nowadays, anti-cytokine biologicals are the top best-selling drugs in the world being used by more than 8 million patients. Indeed, anti-TNF-α treatments alongside other inhibitory antibodies have transformed the pharmacological approach to chronic inflammatory diseases such as rheumatoid arthritis, psoriasis, and inflammatory bowel diseases, all being conditions with increased cardiovascular and cerebrovascular risk [36]. Accordingly, targeting these small intra- and extracellular mediators emerged as a possible strategy to further reduce the risk of acute CV and cerebrovascular (CBV) events in patients under maximal lipid-lowering therapy [145].

This article reviews different biological aspects of cytokines with a specific focus on the pathophysiology of cardiovascular inflammation. Both basic and clinical evidences of the involvement of cytokines in atherosclerosis and its thrombotic complications (i.e., myocardial infarction and ischemic stroke) will be summarized, and major achievements of their specific targeting through inhibitory antibody will be discussed.

## Orchestrating inflammation: cytokine sources and classification

Being produced by nearly every cell type, cytokines are leading effectors of immune responses; however, their effects depend on targeted cell, making them pleiotropic. Downstream signal cascades are indeed differently triggered in different cell types, thus making cytokine effects sometimes paradoxical. Even, some cell types (e.g., macrophages) may express an opposite cytokine/receptor pattern on the same cellular membrane, thus representing the prototypical model of cell polarization as a continuum phenomenon [38, 141]. In addition, cytokine activity is dependent on local environment with different cytokines having similar properties (i.e., redundancy) or showing synergistic effect. Adding a further layer of complexity, the old paradigm of “cytokine storm” is now outdated since both pro- and anti-inflammatory cytokines are in most cases simultaneously released and equally contribute to an effective immune response [117]. The impossibility of discriminating cytokines on the basis of their source or effect has so far determined a somewhat inconsistent nomenclature. A commonly used classification is based on their structure: interleukins (ILs), chemokines, tumor necrosis factors, interferons, transforming, and other growth factors. Sometimes a classification based on their source may be useful, so that the terms adipokines, osteokines, and myokines were coined [121].

Here below, we will summarize the source and general function of cytokines with the most relevant cardiovascular translational perspective.

### Interleukins

**Fig. 1:**
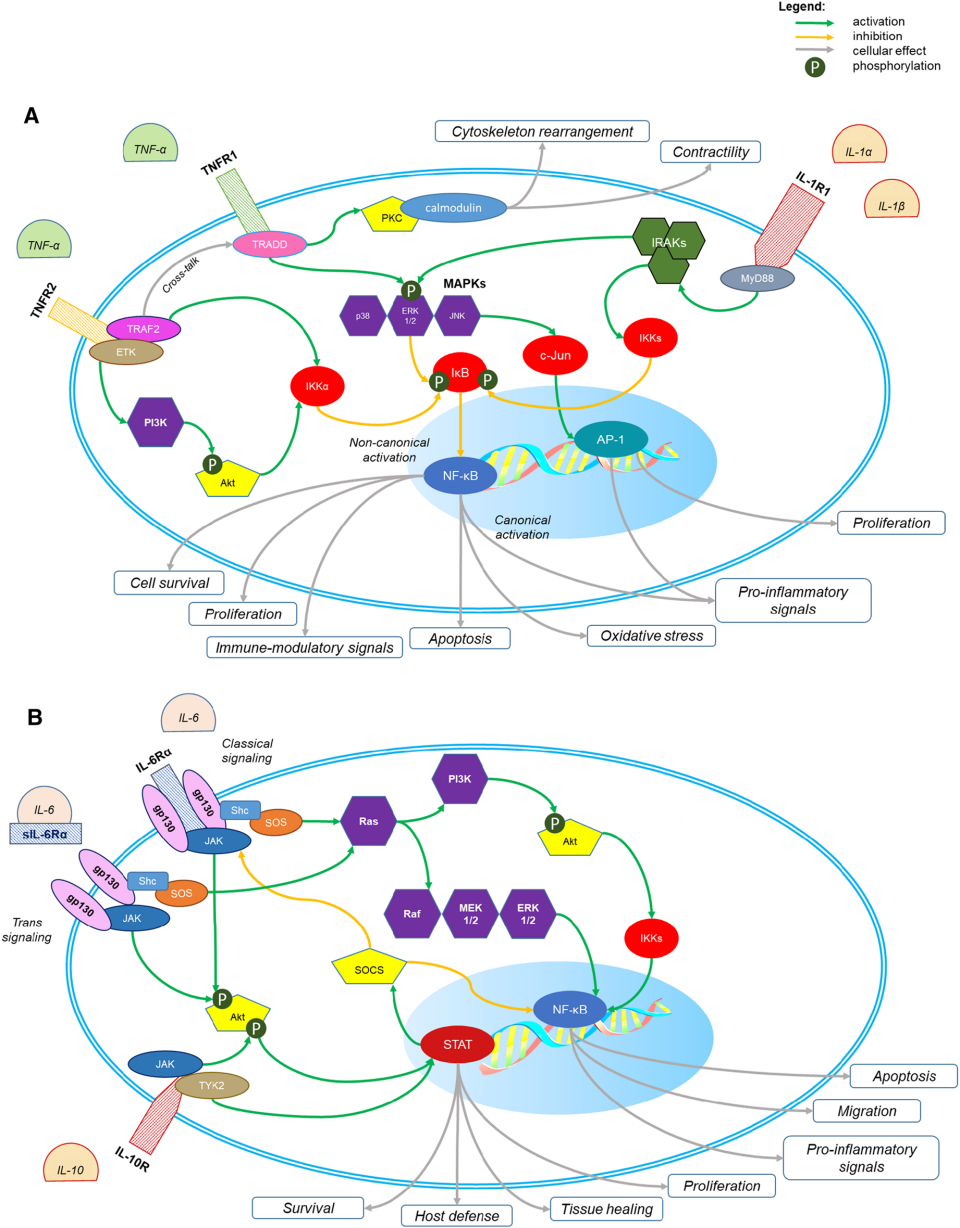
Signaling pathways of the main cytokines involved in cardiovascular diseases. Four main cytokines have been extensively investigated in cardiovascular diseases: tumor necrosis factor α (TNF-α), interleukin 1 (IL-1), IL-6, and IL-10. The intracellular signaling pathways of TNF-α and IL-1 (panel A) converge on two main transcription factors: nuclear factor kappa-light-chain-enhancer of activated B cells (NF-κB) and activator protein 1 (AP-1). Their overall effect is pro-inflammatory. The receptor of TNF-α has two isoforms (TNFR1 and -2). The transduction of signal of TNFR1 is driven by the TNFR1-associated death domain protein (TRADD), which activates the mitogen-activated kinases (MAPKs), namely the extracellular-signal-regulated kinases 1 and 2 (ERK 1/2), the cJun-terminal kinase (JNK), and the p38. In turn, the MAPKs activate c-Jun and phosphorylate the inhibitor of NF-κB (IκB), determining the activation of NF-κB and AP-1. TNFR1 can also activate the protein-kinase C (PKC), that is a Ca^2+^-calmodulin-dependent kinase, that promotes the rearrangement of the cytoskeleton and blunts the contractility of sarcomeres in cardiomyocytes. The transduction of signal of TNFR2 is driven by the TNFR-associated factor 2 (TRAF2) and the endothelial/epithelial tyrosine kinase (ETK). TRAF2 activates the IκB kinase α (IKKα), which phosphorylates IκB and activates NF-κB through the non-canonical pathway, having a final immune-modulatory effect, whereas the canonical pathway leads to apoptosis, increase of oxidative stress, and pro-inflammatory signaling. ETK activates the adaptor protein Akt through the phosphoinositol-3-kinase (PI3K), and eventually activates NF-κB through the non-canonical pathway. IL-1 has two main isoforms, IL-1α and IL-1β, which interact with the same receptor. The intracellular signaling pathway of IL-1 is driven by the myeloid differentiation factor 88 (MyD88), which activates multiple kinases, named interleukin-1-associated kinases (IRAKs), which in turn activate multiple IKKs and the activation of NF-κB through the canonical pathway. The intracellular signaling pathways of IL-6 and IL-10 (panel B) converge on two main transcription factors: NF-κB and the family of signal transducers and activators of transcription (STAT). The overall effect of IL-6 is pro-inflammatory, whereas IL-10 is the main anti-inflammatory cytokine in humans. IL-6 receptor α (IL-6Rα) can be bound to the cell membrane or free in a soluble form (sIL-6Rα). The transduction of signal is initiated by the binding of the receptor with the glycoprotein 130 (gp130), and it may follow two main pathways: the Janus kinase (JAK) pathway and the Ras pathway, through the adaptor proteins Shc. and Son-of-sevenless (SOS). Ras activates the MAPKs’ cascade (namely Raf, MEK 1/2, and ERK 1/2), which activates NF-κB through the canonical pathway. Ras can also activate the PI3K/Akt cascade and NF-κB through the non-canonical pathway. JAK phosphorylates Akt, which in turn promotes migration of STAT inside the nucleus, where it can exert its transcriptional function. The activation of STAT has an overall anti-inflammatory effect, and it inhibits the JAK signaling through a negative feedback mechanism, mediated by the suppressor of cytokine signaling (SOCS). The signaling of IL-10 can be mediated by JAK, through the pathway described above, or through the tyrosine kinase 2 (TYK2). Both pathways converge on STAT

Among them, a special interest toward IL-1 is rising. Forty years after its discovery, the IL-1 family currently counts 11 cytokine members and 10 receptors. They include secretory molecules with agonistic pro-inflammatory activity (IL-1α, IL-1β, IL-18, IL-33, IL-36α, IL-36β, and IL-36γ), receptor antagonists [IL-1 receptor antagonist (Ra), IL-36Ra, and IL-38], and an anti-inflammatory cytokine (IL-37). Especially, IL-1α and β are of cardiovascular interest as inducible cytokines released by monocytes/macrophages, neutrophils, as well as the endothelium and myocardium. However, their biology is quite diverse. Whereas IL-1α is active even as a precursor once released by necrotic cells, IL-1β requires a post-transcriptional activation. The cleavage of pro–IL-1β—and pro–IL-18 as well—into their active forms is largely regulated by the enzymatic activity of caspase-1, ultimately depending on inflammasome activation [6]. Intracellular pathways transducing IL-1 receptor signaling are summarized in Fig. 1.

Also, IL-6 plays a primary role in the inflammatory processes underlying cardiovascular diseases. As an upstream pleiotropic regulator of the inflammatory cascade, IL-6 is secreted by a wide variety of cells (immune cells, chondrocytes, osteoblasts, endothelial cells, skeletal muscle cells, smooth muscle cells, pancreatic islet β-cells, among several others) and exerts several functions ranging from synthesis of acute-phase proteins to activation of endothelial cells, pro-coagulant activity, and stimulation of lymphocyte proliferation/differentiation. Surprisingly, the pro-inflammatory role of IL-6 has been somewhat challenged by evidence of anti-inflammatory and insulin-sensitizing effects [64]. This apparent paradox may reflect the broad range of targeted cells and the influence of intracellular environment, as well as the role of concomitant external stimuli causing an alternate activation of intracellular signaling pathways that may lead to both pro- or anti-inflammatory cellular phenotypes (i.e., trans- vs. classic-signaling, Fig. 1). Interestingly, different biological effects of IL-6 were ascribed to different signal transduction pathways independently of whether IL-6 binds directly to the membrane-bound IL-6R or its soluble form (sIL-6R) with subsequent engagement with the surface glycoprotein (gp)130 [37].

IL-10 is generally considered as an anti-inflammatory cytokine, rising in the late inflammatory phase and facilitating inflammation resolution, tissue clearance, and healing [118]. IL-10 is secreted by Th2 lymphocytes and macrophages as a self-modulating mediator with deactivating properties [53]. IL-10 holds multiple effects eventually downregulating the expression of pro-inflammatory cytokines and reducing immune cell reactivity by blocking NF-kB and blunting the expression of MHC class II antigens and other stimulatory molecules on macrophages (Fig. 1) [244]. IL-10 also facilitates humoral immunity by acting on B-cell survival, proliferation, and antibody synthesis [109].

### Chemokines

Chemokines are small cytokines with chemotactic activity, orchestrating blood cell migration into target tissues. Chemokine classification includes four groups, of which CC and CXC types have important roles in cardiovascular disease.

Of interest, those axes demonstrated to have diverse effects on different molecular pathways, partially due to the fact that they can recognize different ligands. For example, belonging to the C–C motif chemokine ligand (CCL)5/C–C motif chemokine receptor (CCR)5 axis, CCL5 also recognizes different ligands ranging from other chemokines to defensins [8]. This allows CCR5 to influence different systems with both synergistic and modulating effects, and for the same reason, it was considered among the most promising targets for pharmacological interventions [173]. Among other chemokines, CXCL16 is also characterized by a role as a scavenger receptor for oxidized protein, but especially the CXCL12/CXCR4 axis recently attracted the interest for its dual role. Indeed, the beneficial role of CXCL12 on ischemic myocardium (i.e., promotion of angiogenesis and cardiomyocyte protection) and injured endothelium is counterbalanced by a detrimental role of CXC4 in both myocardial recovery and intima preservation [128].

### Tumor necrosis factors

The TNF superfamily includes about 20 ligands and 40 related receptors. The ligands are generally type II transmembrane proteins with a shared amino acid sequence in the binding site. Although some are active in their soluble form, they are usually membrane-bound proteins, and all operate to activate intracellular signal transduction pathways. Depending on their receptor, TNF ligands may have both a pro- and an anti-apoptotic behavior [169] (Fig. 1). The caspase cascade—ultimately leading to apoptosis—may be triggered directly (e.g., death receptor [DR] 4 and 5) or indirectly through the so-called death domains (DD) expressed by some TNFRs (e.g., Fas). Pro-survival properties have been instead reported for DR4, 5 and Fas, TNFR-associated-factors (TRAFs), and decoy receptors acting as competitive inhibitors. This double-edged role is present ubiquitously, with TNFR being expressed in most cell types. Alongside with apoptosis, DD also promotes inflammatory responses through the activation of the nuclear factor kappa-light-chain-enhancer of activated B cell (NF-κB). Under this point of view, it is intuitive how TNF can play a crucial role in cardiovascular health [232].

### Interferons

Among proteins of the interferon (IFN) family, IFN-γ is that of major CV interest as 100–10,000 times more active than other IFN classes. Furthermore, it can modulate up to 2300 human genes, mainly through JAKs–STAT pathway. In its dimeric form, IFN-γ binds two receptors (IFN-γR1 and 2), constitutively associated with downstream activation of JAK 1/2–STAT1 and other JAK-dependent STAT pathways (STAT3, 5b, and others).

IFN-γ is the hallmark of type 1 T helper cell (Th1) activation, the main T-cell subtype involved in cardiovascular disease. The release of IFN-γ activates various cell types and triggers a cascade of cytokines that sustain vascular inflammatory responses: chemoattractant proteins, adhesion molecules, TNF-α, and ILs. Due to extremely wide range of handled genes, pleiotropic activity of IFN-γ also involves detrimental effect on cholesterol transport, characterized by upregulated expression of scavenger receptor and suppressed cholesterol efflux on macrophage and smooth muscle cells [90].

### Growth factors

In humans, the transforming growth factor (TGF) superfamily includes about 30 molecules that may be further classified as TGF-βs (1, 2, and 3), bone morphogenetic proteins, growth differentiation factors, activins, inhibins, nodal, and anti-Mullerian hormone proteins [99]. Among them, especially, TGF-β has a clearly recognized role in cell survival, differentiation, proliferation, and function, and has been implicated in the regulation of inflammatory and reparative responses. Synthetized and secreted as an inactive precursor, latent TGF-β is activated by the interaction with thrombospondin-1 and integrins [99]. Then, active TGF-β exerts its effects by binding to specific receptors (TGF-βRI and II types) that trigger a downstream phosphorylation cascade targeting Smad transcription factors. Mainly involved in fibrotic processes, TGF-β targets several cell types many of which are deeply involved with high cardiovascular relevance: monocytes/macrophages, neutrophils, lymphocytes, fibroblasts, endothelial cells, and myocardial cells [99]. Of growing interest—but without translational implication so far—are the other members of TGF superfamily.

## The role of cytokines in CV and CBV pathology

Recent trials confirmed the long hypothesized “inflammatory theory of atherosclerosis” [145]. Although the atherosclerotic process is most likely to begin with the retention of modified lipoproteins within the vessel wall, soon after monocytes/macrophages and T lymphocytes are recruited by the dysfunctionally activated endothelium and engaged in the vicious circle involving lipids, oxidative stress, and inflammation, eventually leading to the formation of the atherosclerotic plaque necrotic core [138]. The pro-oxidative and pro-inflammatory environment, which is soon established within the vessel wall, is maintained during the whole process of atherogenesis favoring the migration of vascular smooth muscle cells from the media to the intima layer of the artery with collagen deposition and the formation of a fibrous cap isolating the highly pro-thrombotic necrotic core from the blood stream [61]. Not only inflammatory cells play a role in plaque onset and growth, but they also crucially regulate the late catastrophic phases of this process, including atherothrombosis which is the underlying mechanism of most acute cardiac and cerebrovascular ischemic events [25]. Similarly, inflammation drives the evolution of myocardial infarction after ischemia/reperfusion injury, with some degree of intracellular edema appearing already during the ischemic phase, while a rapid and intense extracellular edema characterizes the very early phases of reperfusion. In the hours to follow, a progressive resolution of the edema is observed, which precedes the invasion of myocardial tissue by inflammatory cells including neutrophils and macrophages. Next days are characterized by an intense inflammatory reaction that can have deleterious effects on the tissues, but is also necessary for the replacement of cardiomyocyte debris by collagen and extracellular matrix and the progressive cardiac healing [108]. Similar processes also concern cerebral tissues after an ischemic stroke. Taking into consideration their fundamental role in orchestrating the inflammatory response by regulating cellular trafficking and activation, cytokines have been long investigated as potential therapeutic targets in this context (Table 1).

**Table 1:** Summary of major experimental studies testing cytokine-inhibitory drugs in animal models of acute ischemic cardio- and cerebrovascular diseases

Target	Animal model	Intervention	Phenotype	References
TNF-α	Rabbit; left circumflex coronary artery ligation	Anti-TNF-α Ab	Infarct size reduction	[17, 137]
	Dog; transient closed-chest balloon occlusion of the anterior descending coronary artery	Etanercept	Reduced post-ischemic inflammation and infarct size	[93]
	Dog; permanent coronary artery ligation	Etanercept	Reduced infarct size and malignant ventricular tachyarrhythmias	[250]
	Mouse; permanent coronary artery ligation	TNF‐α inhibitor (CAS1049741‐03‐8)	Worsened left ventricular function	[242]
	Rat; permanent coronary artery ligation	Etanercept	Reduced inflammation, favorable remodeling and preserved ventricular function	[20, 97]
	Mouse; transient coronary artery ligation	Etanercept	Improved cardiac function and reduced infarct size	[77]
	Rat; transient coronary artery ligation	sTNFR-Fc	Reduced infarct size and decreased left ventricular dilation	[231]
	Rat; permanent coronary artery ligation	sTNF-RII	Improved LV end-diastolic pressure and reduced left ventricular dilation	[21]
	Mouse; transient middle cerebral artery occlusion	Anti-TNF-α antibody	Reduced stroke size and preserved post-ischemic blood–brain barrier function	[26]
	Mouse; permanent middle cerebral artery occlusion	Etanercept and sTNF‐α inhibitor	Improved functional outcome with no change in infarct volume	[48]
	Mouse; permanent middle cerebral artery occlusion	TNF binding protein	Decreased infarct volume	[170, 171]
	Rat; transient middle cerebral artery occlusion	Infliximab and etanercept	Decreased infarct volume	[13, 111]
	Rat; transient middle cerebral artery occlusion	Anti-TNF-α antibody and sTNFR1	Decreased infarct volume and edema	[16, 104]
IL-1	Rat; permanent and transient coronary artery ligation Mouse; permanent coronary artery ligation	IL-1Ra	Reduced infarct size and favorable ventricular remodeling	[3, 92]
	Mouse; permanent coronary artery ligation	IL-1 trap	Reduced infarct size and favorable cardiac remodeling	[236]
	Rat; transient coronary artery ligation Mouse; transient coronary artery ligation	IL-1Ra	Reduced infarct size and preserved left ventricular ejection factor	[229]
	Mouse; permanent coronary artery ligation	Anti-IL-1β Ab	Reduced left-ventricular dysfunction, ameliorated myocardial performance and contractile reserve	[227, 228, 254]
	Mouse; transient coronary artery ligation	Anti-IL-1β Ab	Reduced ischemia/reperfusion injury, favorable left- ventricular remodeling, and blunted coronary dysfunction	[101]
	Mouse; transient coronary artery ligation	Anti-IL-1α Ab	Reduced infarct size and preserved left-ventricular function	[159]
	Mouse; transient middle cerebral artery occlusion	Anti-IL-1β Ab	Reduced infarct size and ameliorated post-stroke neurological outcome by BBB preservation	[142]
	Mouse; transient middle cerebral artery occlusion	Anti-IL-1α Ab	Reduced infarct size and ameliorated post-stroke neurological outcome by BBB preservation	[139]
	Mouse; middle cerebral artery photothrombosis	IL-1Ra	Decreased stroke size and post-ischemic BBB damage	[237]
	Rat; permanent middle cerebral artery occlusion	IL-1Ra	Reduced infarct size and ameliorated post-stroke neurological deficit	[79, 191]
	Mouse, permanent and transient middle cerebral artery occlusion	IL-1Ra	Reduced infarct size and ameliorated post-stroke neurological deficit	[160, 233]
	Rat; transient middle cerebral artery occlusion	IL-1Ra	Time- and dose-dependent reduction of infarct size and neurological deficit	[46, 135]
IL-6	Mouse; transient coronary artery ligation	IL-6R blocking Ab	No difference in infarct size, reduce post-ischemic ventricular function	[102]
	Rat; transient coronary artery occlusion	IL6, sIL6R, IL6/sIL6R complex	IL6/sIL6R complex, but not IL6 or sIL6R alone, reduced infarct size	[157]
	Mouse; permanent middle cerebral artery occlusion	sIL-6R	When administered together with IL-6, sIL-6R increased cerebral infarct size	[91]
	Mouse; transient middle cerebral artery occlusion	IL-6Ra	Increased infarct volume and reduced post-stroke neurological function	[247]
	Rat; transient middle cerebral artery occlusion	IL-6	Reduced infarct size	[72]
IL-10	Mouse; permanent coronary artery ligation	IL-10	No difference in infarct size, but decreased ventricular dilation and ameliorated function	[115, 129]
	Mouse; permanent middle cerebral artery occlusion	IL-10	Reduced infarct size	[147]
	Rat; permanent middle cerebral artery occlusion	IL-10	Reduced infarct size	[216]

*Ab* antibody, *LAD* left anterior descending, *IL* interleukin, *(s)IL-6R* (soluble) interleukin 6 receptor, *IL-1R* interleukin 1 receptor, *TNF* tumor necrosis factor, *(s)TNFR* (soluble) tumour necrosis factor receptor

### TNF-α

#### Atherosclerosis

The most extensively studied cytokine in the setting of CV disease remains TNF-α. TNF-α is found in human and experimental atherosclerotic lesions where it is thought to play a role at all stages of plaque development [35, 198]. Indeed, such a cytokine is an important component of particulate debris and soluble substances collected by coronary aspirate during iatrogenic plaque rupture [124]. Of interest, TNF-α from the aspirate was shown to facilitate serotonine-dependent vasoconstriction and to predict saphenous vein bypass graft restenosis risk in diabetic patients [14, 123]. In this sense, data from human coronary aspirate promote a better understanding of the involvement of cytokines to the vulnerable atherosclerotic plaque and may help to find better substances antagonizing microvascular consequences of coronary microembolization, including the no-reflow phenomenon which was reported to be highly regulated by the local inflammatory milieu [78, 124]. Several CV risk factors including obesity, diabetes, aging, and smoking associate with increased circulating levels of this cytokine suggesting its potential role even in the onset of endothelial dysfunction. Indeed, TNFα can directly increase the expression of different pro-inflammatory and pro-coagulant genes [19]. Furthermore, by reducing radical scavengers and facilitating the production of ROS-generating proteins, TNF-α increases vascular oxidative stress [176, 180]. Indeed, by acting on endothelial NO synthase, this cytokine was shown to decrease NO bioavailability and facilitate vascular dysfunction in different animal models and cohorts of patients [42, 180, 248, 251]. Modified lipoproteins were shown to dose-dependently stimulate TNF-α release from different cell types via different intracellular pathways, including the activation of classic pro-inflammatory transcription factors such as AP-1 and NF-kB [114]. Within the vascular wall, TNF-α released in response to oxidized lipids stimulates monocyte differentiation toward the pro-inflammatory macrophages M1 and modulates the expression of several of their enzymes involved in cholesterol metabolism, resulting in increased lipid uptake through acyl-CoA-cholesterol transferase 1, and reduced efflux [141]. As a result, this cytokine facilitates the accumulation of cholesterol in macrophages and foam cells since the earliest stages of atherosclerosis development, thus initiating a vicious circle between macrophage cholesterol uptake and secretion of pro-inflammatory cytokines. Accordingly, atherosclerosis-prone animals deficient for TNFα (double ApoE/TNFα KO) express lower amounts of lipoprotein scavenger receptors and show less fatty streak formation, as compared to ApoE KO littermates when 6 months of age [174, 207]. Of interest, this effect seems to be unrelated to the presence of TNF receptor [174, 207] and is accompanied by blunted levels of several pro-inflammatory mediators including IL-1β, IFNγ, and different adhesion molecules, further proving the deep relationships between cytokines [246]. Formation of foam cell and recruitment of circulating immune cells also contribute to the formation of the plaque’s necrotic core in the later stages of atherosclerosis when TNF-α critically contributes to vascular remodeling. At this stage, TNF-α acts on vascular smooth muscle cells (VSMCs) causing their migration toward the inner layer of the vessel, facilitating their proliferation, and impairing apoptosis: all mechanisms contributing to atheroma progression [57, 86]. Furthermore, during atherosclerosis, VSMCs shift from a physiological contractile phenotype to the so-called synthetic phenotype, more prone to migration and extracellular matrix deposition. TNF-α can prompt such a phenotypic switching, again contributing to atherosclerosis progression [44]. Accordingly, ApoE/TNF-α double KO mice showed reduced vascular wall thickness as compared to ApoE KO littermates and reduced plaque size [29, 127]. The importance of immune cell-derived TNF-α is further demonstrated by an elegant experiment, showing that bone marrow transplantation from animals lacking TNF-α is able to reduce atherosclerosis development in ApoE KO mice [29]. Further data from APOE*3-Leiden transgenic mice demonstrated that TNFα facilitates the progression of lesions toward an advanced phenotype with larger necrotic cores [24]. Similarly, pharmacological inhibition of TNF-α through neutralizing antibodies or treatment with decoy receptors has shown ability to reduce the lesion size in experimental animal models [29].

The latest stages in atherosclerosis are characterized by plaque destabilization, endothelial erosion, and atherothrombosis; of importance, TNF-α has shown effects on all these processes. TNF-α induces the expression of different proteins, e.g., metalloproteinases (MMPs), involved in erosion of the lesion’s fibrous cap at the plaque shoulder [134], yet whether the absence of TNF-α is able to ameliorate outcomes in animal models of plaque destabilization remains to be determined. Furthermore, TNF-α also acts by destabilizing the endothelial layer through the reduction of cell–cell interactions and the formation of intracellular gaps via reducing levels of endothelial cadherin [87, 88]. Also, TNF-α can disassembly actin polymer with structural endothelial changes increasing paracellular passage of macromolecules and disrupting the integrity of the endothelial barrier [11, 87]. Finally, TNF-α dose-dependently reduces endothelial cell viability causing apoptosis through both caspase-dependent and -independent processes [185, 190]. Loss of endothelial integrity favors thrombosis through the exposure of tissue factor in the media layer and the activation of the extrinsic coagulation cascade. Here, again TNF-α is deeply implicated as it activates NF-kB, thus promoting the transcription of tissue factor [81]. Furthermore, this cytokine impairs fibrinolysis by reducing thrombus-resolving tissue plasminogen activator (tPA) via p38MAPK/NF-kB/plasminogen activator inhibitor 1 (PAI-1) [222]. TNF-α also positively modulates thrombosis by increasing the expression of endothelial cell adhesion molecules, facilitating platelet activation and formation of neutrophil extracellular traps (which are known to carry pro-thrombotic factors), stabilizing the fibrin net, and impairing fibrinolysis [182, 184].

#### Myocardial infarction

TNF-α tissue levels are increased in the heart after myocardial infarction (MI). High levels of TNF-α have been shown to suppress cardiac function via impaired calcium signaling, increased inflammation, and oxidative stress [210]. As such, TNF-α has been widely studied as a potential therapeutic target in animal models of cardiac ischemia/reperfusion injury. Antibody-mediated TNF-α neutralization was shown to reduce infarct size in animal models of permanent coronary ligation [17, 93]. Similar results have been obtained by inhibiting TNF-α through the infusion of soluble TNF receptor (sTNFR) 1 that works as a decoy receptor [218]. Conflicting results were obtained, instead, when performing experiments with TNF transgenic or KO animals, showing both reduced and unaltered infarct sizes compared to WT littermates [58, 73, 131, 154]. Similarly, also the use of animals lacking TNF receptors has not led to unambiguous results [131, 167, 189]. These results are most probably due to the onset of compensation mechanisms involving the complex net of interleukins and mediators of inflammation when the gene is constitutionally deleted [96]. Another possible explanation resides in the different effects of TNFR1 and TNFR2, as only the former was able to reduce infarct size when knocked out [73]. Accordingly, the downstream signaling pathways of the two receptors within cardiomyocytes are very different with TNFR1 eliciting NF-kB through ROS and MAPK p38 and JNK while TNFR2 having an overall inhibitory effect on this pro-inflammatory transcription factor [125, 209]. Such a dual role for TNF-α is even more prominent when considering myocardial preconditioning. Indeed, in this context TNF- α was shown to be beneficial, as cardiac accumulation of this cytokine after coronary microembolization reduces infarct size, and such a protective effect is lost after TNF-α signaling disruption [73, 209, 212]. Besides its effect in determining infarct size, TNF-α also contributes to post-MI cardiac dysfunction, remodeling, and onset of heart failure through increased oxidative stress, delayed resolution of inflammation, cardiomyocyte apoptosis, extracellular matrix, and collagen degradation mediated by MMPs, but also adrenoreceptor uncoupling and alteration of mitochondrial function [94, 164]. Again, also in the case of post-ischemic remodeling, TNF-α may have dual effects depending on the receptor, with animals lacking TNFR1 showing blunted contractile dysfunction and inflammation after permanent coronary occlusion, while those without TNFR2 having exaggerated ventricular dilatation, dysfunction, and inflammation [98, 167, 189].

#### Ischemic stroke

Similar to what observed in the heart, TNF-α plays pivotal roles in the pathophysiology of ischemia and ischemia/reperfusion injuries in the brain during ischemic stroke. Here, TNF-α is initially produced after the ischemic insult by activated resident macrophages (i.e., microglia) [41]. Increasing TNF-α levels in the stroke and penumbra areas facilitate the recruitment of circulating inflammatory cells that, once reached the injury site, get activated and in turn release TNF-α, thus promoting a vicious circle involving different pro-inflammatory cytokines [50]. Of interest, TNF-α contributes to the dysfunctional activation of endothelial cells in the blood–brain barrier (BBB), increasing their production of cytokines, ROS, and adhesion molecules, while reducing their barrier function which is a known determinant of stroke outcome [26, 41]. Indeed, TNF-α also up-regulates the expression of different metalloproteinases, including MMP-9 and MMP-3 that are known to digest the BBB regulating tight and adherens junctions such as occludin, claudin-5, and vascular endothelium (VE)-cadherin [26]. Of interest TNF-α, as well as many other inflammatory mediators including MMP-9, has shown also neurotoxic effects, thereby potentially increasing direct neuronal damage [82, 202]. On the other hand, TNF-α also has physiological functions in the brain, including modulation of glutamatergic neurons and regulation of cognitive and behavioral networks [181, 193]. In accordance with its dual role, genetic or pharmacological inhibition of TNF-α signaling yielded conflicting evidence in different experimental models of ischemic stroke. In rodents undergoing permanent transient middle cerebral artery occlusion (pMCAO), global or myeloid cell-specific TNF-α KO is associated with bigger infarct size and worsened post-stroke deficits [49, 132]. Of interest, such protective effects seem to be mediated by the transmembrane form of this cytokine, while its cleavage and systemic release seem to associate with larger lesions [153]. Indeed, pharmacological inhibition of soluble TNF-α via etanercept or specific soluble TNF-α neutralizing proteins associated with improved motor function at 1 and 5 days after pMCAO [48]. Differently from models of permanent ischemia, results from transient MCAO are instead pointing more clearly toward a protective role of anti-TNF-α treatments in ischemia/reperfusion brain injury [26, 148, 219, 245].

### IL-1 family

#### Atherosclerosis

IL-1 family includes a network of 11 cytokines mostly regulating innate immune cells. The role of such mediators in CVD has been studied extensively for 3 of them: IL-1α, IL-1β, and IL-1 receptor antagonist (IL-1Ra, a physiological regulator of IL-1 pathway). IL-1α and β have countless physiological effects known to play a role in the pathogenesis of numerous diseases including atherosclerosis, myocardial infarction, and ischemic stroke [101, 215, 239]. Those cytokines induce the production of several pro-inflammatory mediators, including themselves, in different cell types such as endothelial cells, immune cells, and VSMCs, thereby fueling vascular inflammation through a positive feedback loop mechanism [166, 206, 220]. Of interest, cholesterol crystals are known inducers of inflammasome activation in the atherosclerotic plaque, causing the cleavage of pro-IL-1β and favoring the vascular inflammatory microenvironment [188]. Furthermore, IL-1 affects redox status of tissues by inducing cyclooxygenase 2, prostaglandins, and the inducible form of nitric oxide synthase (iNOS), impairing vasorelaxation, and affecting the anti-thrombotic properties of the endothelium [7, 194, 223]. Furthermore, they directly induce the expression of pro-thrombotic mediators such as tissue factor and plasminogen activator 1 [22, 60] while also facilitating endothelial/ immune cell interactions by increasing levels of different adhesion molecules including ICAM-1, VCAM-1, and P-selectin, as well as the production of chemoattractants such as CCL-2 [33, 149, 224, 241]**.** IL-1 induces the synthesis of platelet-derived growth factor (PDGF) in VSMCs, and facilitates their proliferation and migration [201, 208]. Platelets harbor IL-1α and can enrich their microparticles with IL-1β, thereby facilitating atherothrombosis [30, 103]. As all those processes are known to alter vessel wall homeostasis and accelerate the onset of vascular dysfunction, IL-1α and β are nowadays considered as pro-atherosclerotic mediators and several studies assessed their therapeutic potential in this setting. In murine atherosclerotic models, IL-1 promotes atherosclerosis, while its inhibition reduces plaque burden [63, 120, 163, 239]. In pig vessels, periadventitial injection of IL-1 induces hyperplasia of the media, while IL-1 inhibition can impair intimal thickening in response to damage [168, 211]. Accordingly, hyperlipidemic mice missing IL-1 receptors show reduced arterial remodeling during plaque formation, mainly due to a reduced level of MMPs [9]. Increasing IL-1 signaling by abrogating its physiological inhibitor IL-1Ra results in increased vascular inflammation and structural derangement with the formation of aortic aneurysms [110]. Of interest, a recent elegant study by Libby and coll. disclosed the different contributions of IL-1 isoforms to the progression of atherosclerotic by investigating the effect of specific IL-1α and/or IL-1β antibody-mediated neutralization in ApoE–/– mice early or advanced atherogenesis [239]. Specifically, they reported an important role for IL-1α in arterial remodeling during early experimental atherogenesis, but not in the evolution of established plaques, while IL-1β seemed to drive inflammation and promote the progression to advanced lesions [239]. In this context, we demonstrated that anti-IL-1β neutralizing antibodies could modulate atherothrombosis in animals with systemic inflammation via reducing neutrophil extracellular trap-associated tissue factors [143].

#### Myocardial infarction

IL-1 isoforms participate in cardiac repair after MI, a process initiated by intense inflammatory response and immune cell recruitment to clear damaged cells and predispose extracellular matrix to the reparative phase, characterized by resolution of inflammation, scar formation, and neovascularization [31]. Necrotic and injured cells release danger-associated molecular patterns (DAMPs) that bind to promiscuous receptors known as pattern recognition receptors (PRRs) eventually causing pro-inflammatory activation of resident and recruited immune cells, NLRP3 inflammasome induction, and IL-1β synthesis [2, 107]. Furthermore, dying cells locally release intracellular and membrane-bound IL-1α. As a result, local levels of IL-1 cytokines rise and participate in the propagation of the inflammatory response in ischemic/reperfused myocardium by signaling through MAPK- and NF-κB pathways, activating neighboring and infiltrating cells through IL-1R, and increasing immune cell tissue invasion by increasing myocardial levels of adhesion molecules and chemoattractants [107]. Although important for an effective healing process, excessive inflammatory response associates with adverse cardiac remodeling and worsened post-infarction cardiac function. Indeed, several experimental studies report IL-1 blockade with anakinra or IL-1 trap to modulate ventricular remodeling or infarct size, short time after myocardial infarction [2, 3, 221, 236]. Furthermore, also isoform-specific IL-1 inhibition by mean of anti-IL-1β and anti-IL-1α neutralizing antibodies was shown to reduce inflammasome activation and preserve left-ventricular systolic function [227, 228], even if administered after ischemia [159].

#### Ischemic stroke

IL-1 isoforms are major players in the pathophysiology of ischemic stroke. IL-1β is highly expressed in the brain where it regulates neurotropism and ion channel expression and activity [217, 238]. After an ischemic stroke, IL-1β levels acutely increase mainly due to the activation of local microglia and invading macrophages [56, 142]. Similarly, levels of IL-1α—released in the ischemic brain by injured cells and platelets—raise after 7 days in murine models of cerebral ischemia/reperfusion injury and facilitate neutrophil infiltration and BBB damage [51, 139, 199]. Thus, IL-1 isoforms again play a detrimental role, especially when considering short-term outcome; in line with this notion, mice lacking IL-1α/β expression show reduced post-stroke cerebral damage and neurologic deficit [28]. On the opposite, administration of recombinant IL-1β exacerbated post-stroke neuroinflammation and worsened tMCAO outcome [151]. In keeping with this evidence, pharmacological agents modulating IL-1 signaling showed therapeutic potential in experimental models of stroke [74, 100]. Central and peripheral administration of IL-1Ra ameliorated ischemic brain damage after both tMCAO and pMCAO [51, 152, 191, 233]. Furthermore, we reported that post-ischemic treatment with antibodies neutralizing IL-1α and β reduce stroke size and improve post-stroke neurological deficit in mice undergoing tMCAO, by reducing post-ischemic BBB impairment through different pathways [139, 142]. Specifically, anti-IL-1β antibody reduced neutrophil and MMP-2 activity in ipsilateral hemispheres as compared to vehicle-treated mice, thereby blunting tMCAO-associated vascular endothelial-cadherin reduction [142]. Instead, post-stroke inhibition of IL-1α modulated cerebral injury by blunting endothelial activation and expression of adhesion molecules, thereby reducing penumbral mononuclear phagocyte content and related neurotoxic mediators such as MMPs [139]. Finally, a recent study reported neuroprotective effects of sub-pathological systemic IL-1α doses before brain ischemia [200]. As IL-1α functions locally and circulating levels are hardly detectable both under physiological and pathological conditions, properties of systemic IL-1α remain to be determined.

### IL-6

#### Atherosclerosis

IL-1 is a potent inducer of IL-6 production [230]. IL-6 was recognized as a strong mediator of the acute-phase response, prompting hepatocytes to produce acute-phase reactants, including thrombotic mediators such as fibrinogen and plasminogen activator inhibitor, as well as CRP [205]. Differently from IL-1, IL-6 has shown a dual anti- and pro-inflammatory function, depending on whether the classic or the trans-signaling pathway is activated, and accounting for apparent conflicting results in the literature (reviewed in detail here [203]). Within the vascular tree, IL-6 is produced by endothelial and smooth muscle cells as well as by white blood cells and fibroblast [43, 158, 172]. While exogenous administration of recombinant IL-6 accelerates plaque growth in hyperlipidemic animals [105], its genetic deletion yielded no effect on the early stage of the disease while enhancing plaque progression [204]. Such a late protective effect seems to be mediated by the production of endogenous cytokine antagonists (i.e., IL-1Ra) and anti-inflammatory cytokines, including IL-10 [112, 225].

#### Myocardial infarction

A similar dual effect was confirmed in experimental models of myocardial infarction, where neither IL-6 deletion nor recombinant IL-6 administration affected infarct size, left-ventricular remodeling, or survival in mice after permanent left anterior descending artery (LAD) ligation [75]. Comparably, in mice with I/R injury, weekly injections with an antibody blocking IL-6 receptor did not affect myocardial damage and remodeling as compared to control mice [102]. Recently, a study hypothesized that such neutral effect could result from chest opening and heart puncturing, performed to allow LAD ligation: these procedures would significantly raise the level of cytokines above the effects of I/R-induced cytokine production. Therefore, the authors chose a chest-closed model of I/R injury and compared the effect of transient LAD ligation in WT and IL-6KO animals concluding that, in the absence of major surgical intervention, IL-6 plays a deleterious role in the early phase of reperfusion independently of neutrophil influx, IL-1β, TNFα, tissue factor, and fibrin [113]. Further studies are needed to fully understand what seems to be a “Janus” effect of IL-6 in the development of atherosclerosis and ischemic myocardial injury.

#### Ischemic stroke

Ischemic stroke acutely increases the levels of circulating IL-6 in experimental models, highlighting the potential of this molecule as a therapeutic target. IL-6 is known to act, under physiological conditions, as a neurotrophic factor in the central nervous system. Accordingly, current knowledge suggests IL-6t o be protective in experimental stroke, especially at later time points, when it would support neurogenesis and functional recovery [84]. Yet, apparently contradictory results still generate debates in the field [47]. In accordance with the hypothesis that IL-6 trans-signaling is detrimental, while the classic one may exert protective effects, specific inhibition of trans-signaling mediators (i.e., using the chimeric protein sgp130Fc) may represent an interesting strategy for stroke therapy [69].

### IL-10

#### Atherosclerosis and myocardial infarction

Among the so-called “anti-inflammatory cytokines”, IL-10 is one of the better characterized. IL-10 is expressed in atherosclerotic lesions, where associates with low levels of iNOS and cell death [156]. IL-10 can inhibit different processes involved in plaque progression and rupture such as NF-κB-mediated MMPs, TF, and cyclooxygenase (COX) expression [18, 116, 240]. In keeping with the inflammatory nature of atherosclerosis, pharmacological and genetic modulation of IL-10 pathway revealed important anti-atherogenic effects for this cytokine in different hyperlipidemic experimental models of disease [155, 183, 186]. Similarly, treatment with recombinant IL-10 resulted in inflammation inhibition and reduced ventricular remodeling in animals undergoing permanent LAD ligation via activation of STAT3, reduced MMP-9 activity, and fibrosis [32, 115, 129]. Accordingly, IL-10 deficiency associates with increased production of TNF-α, reduced NO, increased neutrophil recruitment, and myocardial damage after I/R injury [249].

#### Ischemic stroke

As for experimental stroke, also, in this case, the confirmed anti-inflammatory effect of this cytokine has shown important neuroprotective roles. IL-10 overexpressing animals showed reduced short-term infarct volumes and apoptosis after pMCAO [59, 179], while exogenous administration of IL-10 demonstrated therapeutic potentials by limiting post-stroke inflammation [147, 175, 216].

### Other cytokines

IL-4, mainly produced by leukocytes, has shown dual anti- and pro-inflammatory effects and its role in CV diseases remains far from being defined. In animal models of ischemic stroke, exogenous IL-4 administration after tMCAO ameliorated post-stroke motor and behavioral functions, while its effect on stroke size was not always clearly detectable [150, 253]. As for atherosclerosis instead, IL-4 roles remain quite elusive as experiments employing genetic deletion of this cytokine in hyperlipidemic animals showed reduced lesion size and inflammation in IL-4 KO animals [55, 119], while its endogenous administration led to decreased plaque development [106].

IL-17 is produced by different leukocytes as well as endothelial cells. In particular, IL-17 production characterizes a specific CD4 + T helper population known as Th17 cells, expressing STAT3 and involved in different chronic inflammatory diseases including Psoriasis and Crohn disease [27]. Whether and how IL-17 plays a role in atherosclerosis remains controversial. Different studies on atherosclerotic mice using genetic IL-17A deletion, anti-IL-17 antibodies, or inhibiting IL-17 receptor showed its pathogenic effect through increased vessel wall chemoattractant levels and invasion by leukocytes [68, 80, 214, 235]. On the opposite, other studies showed IL-17 to reduce endothelial expression of adhesion molecules with reduced plaque growth [54, 85]. As such, the role of this cytokine on plaque development may depend on different parameters including the stage of atherosclerosis, or the intensity of cytokine modulation. Similar conflicting results were shown by manipulation of IL-23 in atherosclerotic animals. As described for IL-17, also this cytokine is involved in Th17 maintenance and chronic inflammatory disease pathophysiology [27]. Whereas atherosclerotic-prone animals missing IL-23 receptor showed no modulation of atherosclerosis [67], another study recently suggested IL-23 to be protective by reducing proatherogenic gut microbiome and preserving intestinal barrier function [71].

## From bench to bedside: targeting cytokines in CV clinical trials

The possibility to employ immune-suppressing drugs for the prevention of cardiovascular risk raised from the clinical experience in the treatment of chronic inflammatory and autoimmune diseases. As it is well known, these conditions are associated with an increased risk of MACE, and the use of effective treatments, reducing the systemic inflammatory status, was associated with a reduction of the cardiovascular risk up to the reference population levels [36]. The large majority of this evidence has not been collected with targeted RCTs, but it comes from sub-analyses of registration studies, observational cohort studies, and small trials with surrogate outcomes. For instance, the treatment with high-dose methotrexate or cyclosporine for rheumatoid arthritis (RA) is associated with a reduced carotid intima-media thickness and a reduced number of carotid atherosclerotic plaques, compared to the treatment with non-disease-modifying agents [122]. Similarly, the use of the Janus-kinase inhibitor tofacitinib is associated with a reduction of cIMT, despite an increase of circulating LDL-cholesterol [130], whereas the use of the IL-6 inhibitor tocilizumab is associated with an improvement of microvascular endothelial function [197].

Analyses of large registries observed a reduced incidence of MACE in patients with RA or other inflammatory arthritis treated with anti-TNF-α biologic drugs, compared to patients treated with the traditional disease-modifying drugs. However, this supposed benefit of TNF-α inhibition was limited to the duration of the treatment itself instead, was comparable to the benefit obtained using other biologic drugs, and was not observed in patients with a poor response to the anti-TNF-α treatment [196]. Whether reduced cardiovascular events in patients treated with anti-TNF-α drugs are a direct consequence of TNF-α inhibition or an effect of better disease control is still a matter of debate [169], although a recent analysis of the FORWARD registry reported a reduced incidence of CV events in patients treated with TNF-α inhibitors or with the inhibitor of cytotoxic T-lymphocytes-associated protein 4 (CTLA-4) abatacept, compared to the conventional synthetic disease-modifying agents [177].

Nowadays, specific randomized clinical trials (RCTs) have demonstrated that addressing cytokines can be an effective strategy for the prevention of major cardiovascular events in subjects with very-high cardiovascular risk, and for the treatment of complications after acute myocardial ischemia (Table 2). Nonetheless, this strategy is not yet available in clinical practice, mainly because of safety concerns. In this chapter, we will summarize the results of main trials performed up to the present time, focusing on expected benefits and limitations to their translation into “real-life” clinical practice.

**Table 2:** Summary of clinical trials testing cytokine-inhibiting drugs in patients with acute cardiovascular diseases or very-high cardiovascular risk

Drug	Mechanism of action	Trial	Phase	Year	Sample size	Population	Dosage	Efficacy	Safety
Canakinumab	Selective inhibition of IL-1β	CANTOS	3	2017	10,061	Previous MI hs-CRP ≥ 2 mg/L	50 mg, 150 mg and 300 mg every 3 months	↓ hs-CRP ↓ Non-fatal MI/ ischemic stroke or CV death (composite outcome) ↓ HF hospitalization (subgroup analysis) ↑ Functional capacity (LVEF < 50%) ↓ Cancer mortality	↓ Neutrophils ↓ Platelet ↑ Fatal infections
Anakinra	Selective inhibition of IL-1R	VCU-ART3	2	2020	99	STEMI	100 mg/day s.c. or 100 mg bid s.c. for 14 days	↓ hs-CRP ↓ Risk of new-onset HF No effect on LV function Neutral effect on CV events	No increase in infection risk
		Emsley et al	Pilot	2005	34	Ischemic stroke No i.v. thrombolysis	100 mg i.v. loading dose, then 2 mg/kg/h continuous i.v. for 72 h	↓ CRP and IL-6 ↓ 90-day NIHSS and mRS ↓ 90-day death risk	↑ Non-severe infections
		SCIL-STROKE	2	2018	80	Ischemic stroke i.v. thrombolysis	100 mc s.c. bid for 3 days	↓ hs-CRP and IL-6 No ↓ 90-day mRS	Injection site reactions
Tocilizumab	Selective inhibition of IL-6	Kleveland et al	2	2016	117	NSTEMI	280 mg i.v. single dose	↓ hs-CRP and hs-Troponin T Larger effect in patients undergoing PCI	No increase in infection risk
		STAT-MI	2	2017	28	STEMI and NSTEMI	162 mg s.c. single dose	No effect on hs-CRP levels and recurrence of CV events at 30 days Early interruption for expected futility	No increase in infection risk Injection site reactions
Etanercept	Blocking fusion protein anti-TNFα	Padfield et al	Pilot	2013	26	STEMI and NSTEMI	10 mg i.v. single dose	↓ CRP ↑ Platelets aggregation No evaluation of myocardial function or recurrence of cardiovascular events	↑ Risk of HF hospitalization or death

*CRP* C reactive protein, *CV* cardiovascular, *HF* heart failure, *hs* high-sensitivity, *IL* interleukin, *LVEF* left-ventricular ejection fraction, *MI* myocardial infarction, *mRS* modified Rankin scale, *NIHSS* National Institute of Health Stroke Scale, *NSTEMI* non-ST-elevation myocardial infarction, *PCI* percutaneous coronary intervention, *STEMI* ST-elevation myocardial infarction

### Tumor necrosis factor α (TNF-α)

Inhibition of TNF-α signaling is a successful treatment strategy for many inflammatory and autoimmune diseases, such as rheumatoid arthritis (RA), ankylosing spondylitis, and inflammatory bowel diseases. Analyses of large registries observed a reduced incidence of MACE in patients with RA or other inflammatory arthritis treated with anti-TNF-α biologic drugs, compared to patients treated with traditional disease-modifying drugs. As it is well known, inflammatory arthritis is associated per se with a higher risk of MACE, and the risk of patients treated with anti-TNF-α biologic drugs was reduced to the levels of the reference population. However, this supposed benefit of TNF-α inhibition was limited to the duration of the treatment itself instead, was comparable to the benefit obtained using other biologic drugs, and was not observed in patients with a poor response to the anti-TNF-α treatment [196]. Therefore, whether reduced cardiovascular events in patients treated with anti-TNF-α drugs are a direct consequence of TNF-α inhibition or an effect of better disease control is still a matter of debate [169].

The Randomized Etanercept Worldwide Evaluation (RENEWAL) trial combined the results of two trials performed in early 2000s testing efficacy and safety of etanercept, a blocking fusion protein anti-TNF-α, currently employed in the treatment of RA, in patients with chronic heart failure. No evidence of beneficial effects in terms of hospitalization and mortality was found in the etanercept arm [52]; conversely, the phase 2 Anti-TNF Therapy Against Congestive Heart Failure (ATTACH) trial reported an increased risk of hospitalization for heart failure and death for any cause in patients with severe chronic heart failure[45]. In light of this evidence, chronic heart failure is considered a contraindication of anti-TNF-α medications and this represents a strong limitation to their potential use in patients with high cardiovascular risk.

Furthermore, Padfield et al. [178] performed a small placebo-controlled trial, administering etanercept 10 mg within 24 h from hospital admission for myocardial infarction. They observed a significant reduction of CRP and IL-6 circulating levels 24 h after drug administration, alongside an enhancement of platelet activation [178]. In light of this evidence, the existing anti-TNF-α medications are not considered safe enough for a potential use in patients with very-high cardiovascular risk. However, TNF signaling pathways still represent a potential therapeutic target for residual cardiovascular risk; indeed, additional molecular targets belonging to the TNF superfamily, such as Tumor Necrosis Factor-Alpha-Related Apoptosis-Inducing Ligand (TRAIL), Tumor Necrosis Factor-like Weak inducer of apoptosis (TWEAK), and CD40L, are currently under investigation for the treatment of rheumatologic diseases and cancer and the results of ongoing trials are awaited [169]

### Interleukin 1 (IL-1)

IL-1 is the most promising pharmacological target for the reduction of residual risk in patients with very-high cardiovascular risk, after optimization of traditional risk factors, as reported by the over mentioned CANTOS trial [192]. This notorious phase III, placebo-controlled, randomized trial tested the monoclonal antibody anti-IL-1β canakinumab at the dosage of 50 mg, 150 mg and 300 mg every 3 months in subjects with previous myocardial infarction and baseline levels of high-sensitive C reactive protein (hs-CRP) ≥ 2 mg/L. the primary result was the reduction of hs-CRP and IL-6 levels in a dose-dependent manner, associated with a reduced incidence of the composite outcome consisting in non-fatal myocardial infarction, non-fatal stroke, or cardiovascular death for the 150 mg and 300 mg dosages. Separately considering each event, the efficacy was confirmed for myocardial infarction and coronary revascularization only, but not for stroke or cardiovascular death. Noteworthy, the effect was not mediated by any change in circulating lipoproteins. Subsequent post hoc analyses reported that canakinumab 150–300 mg was associated with a reduced risk of hospitalization for heart failure [70], whereas patients with ejection fraction < 50%, treated with canakinumab at any dosage, experienced a significant improvement in aerobic capacity and left-ventricular function [234].

Alongside these promising results, the safety analysis unraveled an increased risk of death for sepsis or infection in patients treated with canakinumab, especially for elderly and diabetic patients. By contrast, a reduced mortality for cancer was associated with the treatment [192], consistent with a large amount of experimental evidence supporting a role of the IL-1 cytokines superfamily in induction and progression of different cancer types [83]. However, all-cause mortality was not affected by the treatment with canakinumab.

Presently, the increased risk of severe infections is the main limitation to the potential application of canakinumab in the treatment of patients with very-high cardiovascular risk. Other common adverse effects reported by the CANTOS in patients receiving canakinumab include neutropenia and thrombocytopenia, not associated with increased bleeding risk [192].

Conflicting results were produced by the phase 2 trials testing the recombinant IL-1 receptor antagonist anakinra, currently approved for the treatment of RA and systemic auto-inflammatory syndromes. In the Virginia Commonwealth University Anakinra Remodeling Trial (VCU-ART), anakinra was administered at the dose of 100 mg/day for 14 days to patients with acute ST Elevated Myocardial Infarction (STEMI), demonstrating a significant reduction of hs-CRP levels 3 months after the event. This reduction was associated with a reduced occurrence of new-onset heart failure, although no significant effect on the left ventricular was demonstrated [5]. Interestingly, no significant incidence of severe infection was associated with the treatment [4]. Nonetheless, it demonstrated also a neutral effect of anakinra on the recurrence of cardiovascular events, defined as a composite outcome of myocardial infarction, unstable angina, ischemic stroke, and symptomatic heart failure [1].

Phase 2 trials employing anakinra have been also performed in patients with acute ischemic stroke. Emsley et al. [66] administered anakinra at a loading dose of 100 mg intravenously within 6 h from the onset of an acute ischemic stroke, followed by a continuous infusion of 2 mg/kg/h for 72 h, reporting a significant reduction of leukocytes, neutrophils, CRP, and IL-6 circulating levels 3 days after the event, associated with a reduction in of disability scores and mortality after 3 months. An increased risk of infections was observed among patients randomized to anakinra, although none was classified as severe. Furthermore, a recent post hoc analysis did not observe an impairment of anti-microbial humoral immunity in patients randomized to anakinra [162]. Unfortunately, the positive effect of anakinra on post-stroke mortality and disability was not confirmed by the subsequent, larger, Subcutaneous Interleukin-1 Receptor Antagonist in Ischemic Stroke (SCIL-STROKE) trial [213], although the significant reduction of IL-6 and CRP circulating levels after 3 days was confirmed. No increased risk of infection was associated with the treatment. Results of these two studies are not comparable, since the SCIL-STROKE trial administered anakinra 100 mg subcutaneously within 6 h from symptom onset, followed by 100 mg every 12 h for 3 days, resulting in a significantly lower administered dose than the previous trial. Furthermore, no patient had undergone intravenous thrombolysis in the first trial, whereas all the enrolled patients in the latter received recombinant Tissue Plasminogen Activator (rTPA). Considering these results, the efficacy and safety of IL-1 receptor blockade for the treatment of ischemic stroke, in addition to intravenous thrombolysis, seems questionable.

### Interleukin 6 (IL-6)

The monoclonal anti-IL-6 antibody tocilizumab—currently approved for the treatment of RA and cytokine release syndrome—was tested in patients with myocardial infarction in a phase 2 trials. Kleveland et al. [126] administered a single dose of tocilizumab 280 mg intravenously prior to coronary angiography to patients with non-ST-elevation myocardial infarction (NSTEMI), reporting a significant decrease of hs-CRP and hs-troponin T compared to placebo, 3 days after the procedure. Interestingly, stronger effects were observed in patients undergoing percutaneous coronary intervention. Conversely, the Short-term Application of Tocilizumab during Myocardial Infarction (STAT-MI) trial [39], testing tocilizumab 162 mg administered subcutaneously within 24 h of hospital admission for myocardial infarction, failed in demonstrating a significant reduction of hs-CRP levels 30 days after administration. This trial was underpowered, since investigators interrupted the recruitment in advance after a futility analysis, revealing a low probability to achieve a significant reduction in major atherosclerotic cardiovascular events. The evident differences between these trials in terms of dosage, time and route of administration, sample size, and timing of outcome measurement impede us from drawing any possible outlook about the efficacy of tocilizumab in patients with acute myocardial infarction. Overall, since none of the trials reported significant adverse events associated with the treatment, investigation on this topic is still ongoing. In particular, the “ASSessing the effect of Anti-IL-6 treatment in MI” (ASSAIL-MI) trial [12] was recently completed, and its results are awaited. Compared to previous trials, the ASSAIL-MI enrolled patients with STEMI and aimed at investigating, as a primary endpoint, the effects of tocilizumab on myocardial salvage, evaluated through cardiac magnetic resonance imaging.

## Future perspectives and conclusions

Advances in the knowledge of cytokines function and the development of innovative therapeutic tools are leading the way for the future application of cytokine modulation in cardiovascular diseases. On one hand, a substantial part of research in this area is focused on identifying new and/or more specific compounds neutralizing the detrimental effects of cytokine activation. IL-1 still represents an interesting target for inhibition: directly through humanized monoclonal antibody alike canakinumab (e.g., gevokizumab [40]) or secondarily induced by inflammasome inhibition [226].

Additional ongoing cytokine-specific approach relies on targeting IL-2 up-regulation, which would exert a beneficial effect through expansion of the Th17 lymphocyte population [252]. A similar and somewhat “classical” approach is also being used in the huge amount of clinical trials targeting chemokines [173] and IFN-γ, whereas presently there is no prospective for clinical translation of TNF/TGF inhibition.

On the other hand, interesting insights would emerge from new approaches, mainly based on post-transcriptional inhibition and the use of nanotechnologies. Cardiovascular medicine is not yet adequately exploring the therapeutic potential of small interfering RNA [136, 187] and nanoparticles [23, 76, 89, 95, 165], as in other research fields, while even more new technologies are emerging, such as the use of CAR T cells [10]. In this regard, the development of tissue-specific technologies of drug delivery could help in reducing the side effects of anti-cytokine treatment, including systemic immune-suppression and the consequent risk of infections. Similarly, future studies will help to understand the optimal duration of the treatment after an acute event, which will be most probably acute rather than chronic to maximize the benefits and reduce side effects.

Finally, the concept of cardiovascular lesions (e.g., atherosclerotic and ischemic) should be redefined: no longer as local disease but rather local chronic foci of systemic inflammation [243]. This shift of paradigm would also help in considering other cytokine circuits with therapeutic potential as those coming from diet/microbioma [71], brain–bone–marrow axis [62], and hypocretin release induced by sleep–wake cycle [161].

The amount of evidence herein reviewed suggests the high potential for immunomodulatory therapies to deeply impact on future CV and CBV pharmacology. Translation from bench to bedside is facilitated by the availability of biologicals specifically interacting with different cytokines and already approved for auto-inflammatory conditions. Future large randomized clinical trials will have the difficult task of finding the balance between the risk of blunted host defense and CV/CBV benefits of anti-cytokine therapies, with the ambitious goal of improving prevention in patients presenting with high risk despite the modern standard of care.

## References

[1] Abbate A, Kontos MC, Abouzaki NA, Melchior RD, Thomas C, Van Tassell BW, Oddi C, Carbone S, Trankle CR, Roberts CS, Mueller GH, Gambill ML, Christopher S, Markley R, Vetrovec GW, Dinarello CA, Biondi-Zoccai G (2015). Comparative safety of interleukin-1 blockade with anakinra in patients with ST-segment elevation acute myocardial infarction (from the VCU-ART and VCU-ART2 pilot studies). Am J Cardiol.

[2] Abbate A, Salloum FN, Van Tassell BW, Vecile E, Toldo S, Seropian I, Mezzaroma E, Dobrina A (2011). Alterations in the interleukin-1/interleukin-1 receptor antagonist balance modulate cardiac remodeling following myocardial infarction in the mouse. PLoS ONE.

[3] Abbate A, Salloum FN, Vecile E, Das A, Hoke NN, Straino S, Biondi-Zoccai GG, Houser JE, Qureshi IZ, Ownby ED, Gustini E, Biasucci LM, Severino A, Capogrossi MC, Vetrovec GW, Crea F, Baldi A, Kukreja RC, Dobrina A (2008). Anakinra, a recombinant human interleukin-1 receptor antagonist, inhibits apoptosis in experimental acute myocardial infarction. Circulation.

[4] Abbate A, Trankle CR, Buckley LF, Lipinski MJ, Appleton D, Kadariya D, Canada JM, Carbone S, Roberts CS, Abouzaki N, Melchior R, Christopher S, Turlington J, Mueller G, Garnett J, Thomas C, Markley R, Wohlford GF, Puckett L, Medina de Chazal H, Chiabrando JG, Bressi E, Del Buono MG, Schatz A, Vo C, Dixon DL, Biondi-Zoccai GG, Kontos MC, Van Tassell BW (2020). Interleukin-1 blockade inhibits the acute inflammatory response in patients with ST-segment-elevation myocardial infarction. J Am Heart Assoc.

[5] Abbate A, Van Tassell BW, Biondi-Zoccai G, Kontos MC, Grizzard JD, Spillman DW, Oddi C, Roberts CS, Melchior RD, Mueller GH, Abouzaki NA, Rengel LR, Varma A, Gambill ML, Falcao RA, Voelkel NF, Dinarello CA, Vetrovec GW (2013). Effects of interleukin-1 blockade with anakinra on adverse cardiac remodeling and heart failure after acute myocardial infarction [from the Virginia Commonwealth University-Anakinra Remodeling Trial (2) (VCU-ART2) pilot study]. Am J Cardiol.

[6] Agostini L, Martinon F, Burns K, McDermott MF, Hawkins PN, Tschopp J (2004). NALP3 forms an IL-1beta-processing inflammasome with increased activity in Muckle-Wells autoinflammatory disorder. Immunity.

[7] Akarasereenont P, Techatrisak K, Chotewuttakorn S, Thaworn A (1999). The induction of cyclooxygenase-2 in IL-1beta-treated endothelial cells is inhibited by prostaglandin E2 through cAMP. Mediators Inflamm.

[8] Alard JE, Ortega-Gomez A, Wichapong K, Bongiovanni D, Horckmans M, Megens RT, Leoni G, Ferraro B, Rossaint J, Paulin N, Ng J, Ippel H, Suylen D, Hinkel R, Blanchet X, Gaillard F, D’Amico M, von Hundelshausen P, Zarbock A, Scheiermann C, Hackeng TM, Steffens S, Kupatt C, Nicolaes GA, Weber C, Soehnlein O (2015). Recruitment of classical monocytes can be inhibited by disturbing heteromers of neutrophil HNP1 and platelet CCL5. Sci Transl Med.

[9] Alexander MR, Moehle CW, Johnson JL, Yang Z, Lee JK, Jackson CL, Owens GK (2012). Genetic inactivation of IL-1 signaling enhances atherosclerotic plaque instability and reduces outward vessel remodeling in advanced atherosclerosis in mice. J Clin Invest.

[10] Amor C, Feucht J, Leibold J, Ho YJ, Zhu C, Alonso-Curbelo D, Mansilla-Soto J, Boyer JA, Li X, Giavridis T, Kulick A, Houlihan S, Peerschke E, Friedman SL, Ponomarev V, Piersigilli A, Sadelain M, Lowe SW (2020). Senolytic CAR T cells reverse senescence-associated pathologies. Nature.

[11] Angelini DJ, Hyun SW, Grigoryev DN, Garg P, Gong P, Singh IS, Passaniti A, Hasday JD, Goldblum SE (2006). TNF-alpha increases tyrosine phosphorylation of vascular endothelial cadherin and opens the paracellular pathway through fyn activation in human lung endothelia. Am J Physiol Lung Cell Mol Physiol.

[12] Anstensrud AK, Woxholt S, Sharma K, Broch K, Bendz B, Aakhus S, Ueland T, Amundsen BH, Damas JK, Hopp E, Kleveland O, Stensaeth KH, Opdahl A, Klow NE, Seljeflot I, Andersen GO, Wiseth R, Aukrust P, Gullestad L (2019). Rationale for the ASSAIL-MI-trial: a randomised controlled trial designed to assess the effect of tocilizumab on myocardial salvage in patients with acute ST-elevation myocardial infarction (STEMI). Open Heart.

[13] Arango-Davila CA, Vera A, Londono AC, Echeverri AF, Canas F, Cardozo CF, Orozco JL, Rengifo J, Canas CA (2015). Soluble or soluble/membrane TNF-alpha inhibitors protect the brain from focal ischemic injury in rats. Int J Neurosci.

[14] Baars T, Konorza T, Kahlert P, Mohlenkamp S, Erbel R, Heusch G, Kleinbongard P (2013). Coronary aspirate TNFalpha reflects saphenous vein bypass graft restenosis risk in diabetic patients. Cardiovasc Diabetol.

[15] Bahrami A, Liberale L, Reiner Z, Carbone F, Montecucco F, Sahebkar A (2020). Inflammatory biomarkers for cardiovascular risk stratification in familial hypercholesterolemia. Rev Physiol Biochem Pharmacol.

[16] Barone FC, Arvin B, White RF, Miller A, Webb CL, Willette RN, Lysko PG, Feuerstein GZ (1997). Tumor necrosis factor-alpha. A mediator of focal ischemic brain injury. Stroke.

[17] Belosjorow S, Bolle I, Duschin A, Heusch G, Schulz R (2003). TNF-alpha antibodies are as effective as ischemic preconditioning in reducing infarct size in rabbits. Am J Physiol Heart Circ Physiol.

[18] Berg DJ, Zhang J, Lauricella DM, Moore SA (2001). Il-10 is a central regulator of cyclooxygenase-2 expression and prostaglandin production. J Immunol.

[19] Bergh N, Ulfhammer E, Glise K, Jern S, Karlsson L (2009). Influence of TNF-alpha and biomechanical stress on endothelial anti- and prothrombotic genes. Biochem Biophys Res Commun.

[20] Berry MF, Woo YJ, Pirolli TJ, Bish LT, Moise MA, Burdick JW, Morine KJ, Jayasankar V, Gardner TJ, Sweeney HL (2004). Administration of a tumor necrosis factor inhibitor at the time of myocardial infarction attenuates subsequent ventricular remodeling. J Heart Lung Transplant.

[21] Berthonneche C, Sulpice T, Boucher F, Gouraud L, de Leiris J, O'Connor SE, Herbert JM, Janiak P (2004). New insights into the pathological role of TNF-alpha in early cardiac dysfunction and subsequent heart failure after infarction in rats. Am J Physiol Heart Circ Physiol.

[22] Bevilacqua MP, Pober JS, Majeau GR, Cotran RS, Gimbrone MA (1984). Interleukin 1 (IL-1) induces biosynthesis and cell surface expression of procoagulant activity in human vascular endothelial cells. J Exp Med.

[23] Boada C, Zinger A, Tsao C, Zhao P, Martinez JO, Hartman K, Naoi T, Sukhoveshin R, Sushnitha M, Molinaro R, Trachtenberg B, Cooke JP, Tasciotti E (2020). Rapamycin-loaded biomimetic nanoparticles reverse vascular inflammation. Circ Res.

[24] Boesten LS, Zadelaar AS, van Nieuwkoop A, Gijbels MJ, de Winther MP, Havekes LM, van Vlijmen BJ (2005). Tumor necrosis factor-alpha promotes atherosclerotic lesion progression in APOE*3-Leiden transgenic mice. Cardiovasc Res.

[25] Bonaventura A, Liberale L, Carbone F, Vecchie A, Diaz-Canestro C, Camici GG, Montecucco F, Dallegri F (2018). The pathophysiological role of neutrophil extracellular traps in inflammatory diseases. Thromb Haemost.

[26] Bonetti NR, Diaz-Canestro C, Liberale L, Crucet M, Akhmedov A, Merlini M, Reiner MF, Gobbato S, Stivala S, Kollias G, Ruschitzka F, Luscher TF, Beer JH, Camici GG (2019). Tumour necrosis factor-alpha inhibition improves stroke outcome in a mouse model of rheumatoid arthritis. Sci Rep.

[27] Boniface K, Blom B, Liu YJ, de Waal MR (2008). From interleukin-23 to T-helper 17 cells: human T-helper cell differentiation revisited. Immunol Rev.

[28] Boutin H, LeFeuvre RA, Horai R, Asano M, Iwakura Y, Rothwell NJ (2001). Role of IL-1alpha and IL-1beta in ischemic brain damage. J Neurosci.

[29] Branen L, Hovgaard L, Nitulescu M, Bengtsson E, Nilsson J, Jovinge S (2004). Inhibition of tumor necrosis factor-alpha reduces atherosclerosis in apolipoprotein E knockout mice. Arterioscler Thromb Vasc Biol.

[30] Brown GT, McIntyre TM (2011). Lipopolysaccharide signaling without a nucleus: kinase cascades stimulate platelet shedding of proinflammatory IL-1beta-rich microparticles. J Immunol.

[31] Bujak M, Dobaczewski M, Chatila K, Mendoza LH, Li N, Reddy A, Frangogiannis NG (2008). Interleukin-1 receptor type I signaling critically regulates infarct healing and cardiac remodeling. Am J Pathol.

[32] Burchfield JS, Iwasaki M, Koyanagi M, Urbich C, Rosenthal N, Zeiher AM, Dimmeler S (2008). Interleukin-10 from transplanted bone marrow mononuclear cells contributes to cardiac protection after myocardial infarction. Circ Res.

[33] Burne MJ, Elghandour A, Haq M, Saba SR, Norman J, Condon T, Bennett F, Rabb H (2001). IL-1 and TNF independent pathways mediate ICAM-1/VCAM-1 up-regulation in ischemia reperfusion injury. J Leukoc Biol.

[34] Camici GG, Liberale L (2017). Aging: the next cardiovascular disease?. Eur Heart J.

[35] Canault M, Peiretti F, Poggi M, Mueller C, Kopp F, Bonardo B, Bastelica D, Nicolay A, Alessi MC, Nalbone G (2008). Progression of atherosclerosis in ApoE-deficient mice that express distinct molecular forms of TNF-alpha. J Pathol.

[36] Carbone F, Bonaventura A, Liberale L, Paolino S, Torre F, Dallegri F, Montecucco F, Cutolo M (2020). Atherosclerosis in rheumatoid arthritis: promoters and opponents. Clin Rev Allergy Immunol.

[37] Carbone F, Liberale L, Bonaventura A, Cea M, Montecucco F (2016). Targeting inflammation in primary cardiovascular prevention. Curr Pharm Des.

[38] Carbone F, Satta N, Burger F, Roth A, Lenglet S, Pagano S, Lescuyer P, Bertolotto M, Spinella G, Pane B, Palombo D, Pende A, Dallegri F, Mach F, Vuilleumier N, Montecucco F (2016). Vitamin D receptor is expressed within human carotid plaques and correlates with pro-inflammatory M1 macrophages. Vascul Pharmacol.

[39] Carroll MB, Haller C, Smith C (2018). Short-term application of tocilizumab during myocardial infarction (STAT-MI). Rheumatol Int.

[40] Cavelti-Weder C, Babians-Brunner A, Keller C, Stahel MA, Kurz-Levin M, Zayed H, Solinger AM, Mandrup-Poulsen T, Dinarello CA, Donath MY (2012). Effects of gevokizumab on glycemia and inflammatory markers in type 2 diabetes. Diabetes Care.

[41] Chen AQ, Fang Z, Chen XL, Yang S, Zhou YF, Mao L, Xia YP, Jin HJ, Li YN, You MF, Wang XX, Lei H, He QW, Hu B (2019). Microglia-derived TNF-alpha mediates endothelial necroptosis aggravating blood brain-barrier disruption after ischemic stroke. Cell Death Dis.

[42] Chen X, Andresen BT, Hill M, Zhang J, Booth F, Zhang C (2008). Role of reactive oxygen species in tumor necrosis factor-alpha induced endothelial dysfunction. Curr Hypertens Rev.

[43] Chi L, Li Y, Stehno-Bittel L, Gao J, Morrison DC, Stechschulte DJ, Dileepan KN (2001). Interleukin-6 production by endothelial cells via stimulation of protease-activated receptors is amplified by endotoxin and tumor necrosis factor-alpha. J Interferon Cytokine Res.

[44] Choi S, Park M, Kim J, Park W, Kim S, Lee DK, Hwang JY, Choe J, Won MH, Ryoo S, Ha KS, Kwon YG, Kim YM (2018). TNF-alpha elicits phenotypic and functional alterations of vascular smooth muscle cells by miR-155-5p-dependent down-regulation of cGMP-dependent kinase 1. J Biol Chem.

[45] Chung ES, Packer M, Lo KH, Fasanmade AA, Willerson JT, Anti TNFTACHFI (2003). Randomized, double-blind, placebo-controlled, pilot trial of infliximab, a chimeric monoclonal antibody to tumor necrosis factor-alpha, in patients with moderate-to-severe heart failure: results of the anti-TNF Therapy Against Congestive Heart Failure (ATTACH) trial. Circulation.

[46] Clark SR, McMahon CJ, Gueorguieva I, Rowland M, Scarth S, Georgiou R, Tyrrell PJ, Hopkins SJ, Rothwell NJ (2008). Interleukin-1 receptor antagonist penetrates human brain at experimentally therapeutic concentrations. J Cereb Blood Flow Metab.

[47] Clark WM, Rinker LG, Lessov NS, Hazel K, Hill JK, Stenzel-Poore M, Eckenstein F (2000). Lack of interleukin-6 expression is not protective against focal central nervous system ischemia. Stroke.

[48] Clausen BH, Degn M, Martin NA, Couch Y, Karimi L, Ormhoj M, Mortensen ML, Gredal HB, Gardiner C, Sargent II, Szymkowski DE, Petit GH, Deierborg T, Finsen B, Anthony DC, Lambertsen KL (2014). Systemically administered anti-TNF therapy ameliorates functional outcomes after focal cerebral ischemia. J Neuroinflammation.

[49] Clausen BH, Degn M, Sivasaravanaparan M, Fogtmann T, Andersen MG, Trojanowsky MD, Gao H, Hvidsten S, Baun C, Deierborg T, Finsen B, Kristensen BW, Bak ST, Meyer M, Lee J, Nedospasov SA, Brambilla R, Lambertsen KL (2016). Conditional ablation of myeloid TNF increases lesion volume after experimental stroke in mice, possibly via altered ERK1/2 signaling. Sci Rep.

[50] Clausen BH, Lambertsen KL, Babcock AA, Holm TH, Dagnaes-Hansen F, Finsen B (2008). Interleukin-1beta and tumor necrosis factor-alpha are expressed by different subsets of microglia and macrophages after ischemic stroke in mice. J Neuroinflammation.

[51] Clausen BH, Lambertsen KL, Dagnaes-Hansen F, Babcock AA, von Linstow CU, Meldgaard M, Kristensen BW, Deierborg T, Finsen B (2016). Cell therapy centered on IL-1Ra is neuroprotective in experimental stroke. Acta Neuropathol.

[52] Coletta AP, Clark AL, Banarjee P, Cleland JG (2002). Clinical trials update: RENEWAL (RENAISSANCE and RECOVER) and ATTACH. Eur J Heart Fail.

[53] Coomes SM, Kannan Y, Pelly VS, Entwistle LJ, Guidi R, Perez-Lloret J, Nikolov N, Müller W, Wilson MS (2017). CD4(+) Th2 cells are directly regulated by IL-10 during allergic airway inflammation. Mucosal Immunol.

[54] Danzaki K, Matsui Y, Ikesue M, Ohta D, Ito K, Kanayama M, Kurotaki D, Morimoto J, Iwakura Y, Yagita H, Tsutsui H, Uede T (2012). Interleukin-17A deficiency accelerates unstable atherosclerotic plaque formation in apolipoprotein E-deficient mice. Arterioscler Thromb Vasc Biol.

[55] Davenport P, Tipping PG (2003). The role of interleukin-4 and interleukin-12 in the progression of atherosclerosis in apolipoprotein E-deficient mice. Am J Pathol.

[56] Davies CA, Loddick SA, Toulmond S, Stroemer RP, Hunt J, Rothwell NJ (1999). The progression and topographic distribution of interleukin-1beta expression after permanent middle cerebral artery occlusion in the rat. J Cereb Blood Flow Metab.

[57] Davis R, Pillai S, Lawrence N, Sebti S, Chellappan SP (2012). TNF-alpha-mediated proliferation of vascular smooth muscle cells involves Raf-1-mediated inactivation of Rb and transcription of E2F1-regulated genes. Cell Cycle.

[58] Dawn B, Guo Y, Rezazadeh A, Wang OL, Stein AB, Hunt G, Varma J, Xuan YT, Wu WJ, Tan W, Zhu X, Bolli R (2004). Tumor necrosis factor-alpha does not modulate ischemia/reperfusion injury in naive myocardium but is essential for the development of late preconditioning. J Mol Cell Cardiol.

[59] de Bilbao F, Arsenijevic D, Moll T, Garcia-Gabay I, Vallet P, Langhans W, Giannakopoulos P (2009). In vivo over-expression of interleukin-10 increases resistance to focal brain ischemia in mice. J Neurochem.

[60] Dong J, Fujii S, Imagawa S, Matsumoto S, Matsushita M, Todo S, Tsutsui H, Sobel BE (2007). IL-1 and IL-6 induce hepatocyte plasminogen activator inhibitor-1 expression through independent signaling pathways converging on C/EBPdelta. Am J Physiol Cell Physiol.

[61] Doran AC, Meller N, McNamara CA (2008). Role of smooth muscle cells in the initiation and early progression of atherosclerosis. Arterioscler Thromb Vasc Biol.

[62] Dutta P, Courties G, Wei Y, Leuschner F, Gorbatov R, Robbins CS, Iwamoto Y, Thompson B, Carlson AL, Heidt T, Majmudar MD, Lasitschka F, Etzrodt M, Waterman P, Waring MT, Chicoine AT, van der Laan AM, Niessen HW, Piek JJ, Rubin BB, Butany J, Stone JR, Katus HA, Murphy SA, Morrow DA, Sabatine MS, Vinegoni C, Moskowitz MA, Pittet MJ, Libby P, Lin CP, Swirski FK, Weissleder R, Nahrendorf M (2012). Myocardial infarction accelerates atherosclerosis. Nature.

[63] Elhage R, Maret A, Pieraggi MT, Thiers JC, Arnal JF, Bayard F (1998). Differential effects of interleukin-1 receptor antagonist and tumor necrosis factor binding protein on fatty-streak formation in apolipoprotein E-deficient mice. Circulation.

[64] Ellingsgaard H, Hauselmann I, Schuler B, Habib AM, Baggio LL, Meier DT, Eppler E, Bouzakri K, Wueest S, Muller YD, Hansen AM, Reinecke M, Konrad D, Gassmann M, Reimann F, Halban PA, Gromada J, Drucker DJ, Gribble FM, Ehses JA, Donath MY (2011). Interleukin-6 enhances insulin secretion by increasing glucagon-like peptide-1 secretion from L cells and alpha cells. Nat Med.

[65] Elliott MJ, Maini RN, Feldmann M, Long-Fox A, Charles P, Katsikis P, Brennan FM, Walker J, Bijl H, Ghrayeb J (1993). Treatment of rheumatoid arthritis with chimeric monoclonal antibodies to tumor necrosis factor alpha. Arthritis Rheum.

[66] Emsley HC, Smith CJ, Georgiou RF, Vail A, Hopkins SJ, Rothwell NJ, Tyrrell PJ, Acute Stroke I (2005). A randomised phase II study of interleukin-1 receptor antagonist in acute stroke patients. J Neurol Neurosurg Psychiatry.

[67] Engelbertsen D, Depuydt MAC, Verwilligen RAF, Rattik S, Levinsohn E, Edsfeldt A, Kuperwaser F, Jarolim P, Lichtman AH (2018). IL-23R deficiency does not impact atherosclerotic plaque development in mice. J Am Heart Assoc.

[68] Erbel C, Chen L, Bea F, Wangler S, Celik S, Lasitschka F, Wang Y, Bockler D, Katus HA, Dengler TJ (2009). Inhibition of IL-17A attenuates atherosclerotic lesion development in apoE-deficient mice. J Immunol.

[69] Erta M, Quintana A, Hidalgo J (2012). Interleukin-6, a major cytokine in the central nervous system. Int J Biol Sci.

[70] Everett BM, Cornel JH, Lainscak M, Anker SD, Abbate A, Thuren T, Libby P, Glynn RJ, Ridker PM (2019). Anti-inflammatory therapy with canakinumab for the prevention of hospitalization for heart failure. Circulation.

[71] Fatkhullina AR, Peshkova IO, Dzutsev A, Aghayev T, McCulloch JA, Thovarai V, Badger JH, Vats R, Sundd P, Tang HY, Kossenkov AV, Hazen SL, Trinchieri G, Grivennikov SI, Koltsova EK (2018). An interleukin-23-interleukin-22 axis regulates intestinal microbial homeostasis to protect from diet-induced atherosclerosis. Immunity.

[72] Feng Q, Wang YI, Yang Y (2015). Neuroprotective effect of interleukin-6 in a rat model of cerebral ischemia. Exp Ther Med.

[73] Flaherty MP, Guo Y, Tiwari S, Rezazadeh A, Hunt G, Sanganalmath SK, Tang XL, Bolli R, Dawn B (2008). The role of TNF-alpha receptors p55 and p75 in acute myocardial ischemia/reperfusion injury and late preconditioning. J Mol Cell Cardiol.

[74] Friedlander RM, Gagliardini V, Hara H, Fink KB, Li W, MacDonald G, Fishman MC, Greenberg AH, Moskowitz MA, Yuan J (1997). Expression of a dominant negative mutant of interleukin-1 beta converting enzyme in transgenic mice prevents neuronal cell death induced by trophic factor withdrawal and ischemic brain injury. J Exp Med.

[75] Fuchs M, Hilfiker A, Kaminski K, Hilfiker-Kleiner D, Guener Z, Klein G, Podewski E, Schieffer B, Rose-John S, Drexler H (2003). Role of interleukin-6 for LV remodeling and survival after experimental myocardial infarction. FASEB J.

[76] Gao C, Huang Q, Liu C, Kwong CHT, Yue L, Wan JB, Lee SMY, Wang R (2020). Treatment of atherosclerosis by macrophage-biomimetic nanoparticles via targeted pharmacotherapy and sequestration of proinflammatory cytokines. Nat Commun.

[77] Gao C, Liu Y, Yu Q, Yang Q, Li B, Sun L, Yan W, Cai X, Gao E, Xiong L, Wang H, Tao L (2015). TNF-alpha antagonism ameliorates myocardial ischemia-reperfusion injury in mice by upregulating adiponectin. Am J Physiol Heart Circ Physiol.

[78] Gao XM, Su Y, Moore S, Han LP, Kiriazis H, Lu Q, Zhao WB, Ruze A, Fang BB, Duan MJ, Du XJ (2019). Relaxin mitigates microvascular damage and inflammation following cardiac ischemia-reperfusion. Basic Res Cardiol.

[79] Garcia JH, Liu KF, Relton JK (1995). Interleukin-1 receptor antagonist decreases the number of necrotic neurons in rats with middle cerebral artery occlusion. Am J Pathol.

[80] Ge S, Hertel B, Koltsova EK, Sorensen-Zender I, Kielstein JT, Ley K, Haller H, von Vietinghoff S (2013). Increased atherosclerotic lesion formation and vascular leukocyte accumulation in renal impairment are mediated by interleukin-17A. Circ Res.

[81] Gebhard C, Stampfli SF, Gebhard CE, Akhmedov A, Breitenstein A, Camici GG, Holy EW, Luscher TF, Tanner FC (2009). Guggulsterone, an anti-inflammatory phytosterol, inhibits tissue factor and arterial thrombosis. Basic Res Cardiol.

[82] Gelbard HA, Dzenko KA, DiLoreto D, del Cerro C, del Cerro M, Epstein LG (1993). Neurotoxic effects of tumor necrosis factor alpha in primary human neuronal cultures are mediated by activation of the glutamate AMPA receptor subtype: implications for AIDS neuropathogenesis. Dev Neurosci.

[83] Gelfo V, Romaniello D, Mazzeschi M, Sgarzi M, Grilli G, Morselli A, Manzan B, Rihawi K, Lauriola M (2020). Roles of IL-1 in cancer: from tumor progression to resistance to targeted therapies. Int J Mol Sci.

[84] Gertz K, Kronenberg G, Kalin RE, Baldinger T, Werner C, Balkaya M, Eom GD, Hellmann-Regen J, Krober J, Miller KR, Lindauer U, Laufs U, Dirnagl U, Heppner FL, Endres M (2012). Essential role of interleukin-6 in post-stroke angiogenesis. Brain.

[85] Gistera A, Robertson AK, Andersson J, Ketelhuth DF, Ovchinnikova O, Nilsson SK, Lundberg AM, Li MO, Flavell RA, Hansson GK (2013). Transforming growth factor-beta signaling in T cells promotes stabilization of atherosclerotic plaques through an interleukin-17-dependent pathway. Sci Transl Med.

[86] Goetze S, Xi XP, Kawano Y, Kawano H, Fleck E, Hsueh WA, Law RE (1999). TNF-alpha-induced migration of vascular smooth muscle cells is MAPK dependent. Hypertension.

[87] Goldblum SE, Ding X, Campbell-Washington J (1993). TNF-alpha induces endothelial cell F-actin depolymerization, new actin synthesis, and barrier dysfunction. Am J Physiol.

[88] Goldblum SE, Sun WL (1990). Tumor necrosis factor-alpha augments pulmonary arterial transendothelial albumin flux in vitro. Am J Physiol.

[89] Gorabi AM, Kiaie N, Reiner Z, Carbone F, Montecucco F, Sahebkar A (2019). The therapeutic potential of nanoparticles to reduce inflammation in atherosclerosis. Biomolecules.

[90] Grewal T, Priceputu E, Davignon J, Bernier L (2001). Identification of a gamma-interferon-responsive element in the promoter of the human macrophage scavenger receptor A gene. Arterioscler Thromb Vasc Biol.

[91] Gronhoj MH, Clausen BH, Fenger CD, Lambertsen KL, Finsen B (2017). Beneficial potential of intravenously administered IL-6 in improving outcome after murine experimental stroke. Brain Behav Immun.

[92] Grothusen C, Hagemann A, Attmann T, Braesen J, Broch O, Cremer J, Schoettler J (2012). Impact of an interleukin-1 receptor antagonist and erythropoietin on experimental myocardial ischemia/reperfusion injury. Sci World J.

[93] Gu Q, Yang XP, Bonde P, DiPaula A, Fox-Talbot K, Becker LC (2006). Inhibition of TNF-alpha reduces myocardial injury and proinflammatory pathways following ischemia-reperfusion in the dog. J Cardiovasc Pharmacol.

[94] Gulick T, Chung MK, Pieper SJ, Lange LG, Schreiner GF (1989). Interleukin 1 and tumor necrosis factor inhibit cardiac myocyte beta-adrenergic responsiveness. Proc Natl Acad Sci USA.

[95] Guo J, Li D, Tao H, Li G, Liu R, Dou Y, Jin T, Li L, Huang J, Hu H, Zhang J (2019). Cyclodextrin-derived intrinsically bioactive nanoparticles for treatment of acute and chronic inflammatory diseases. Adv Mater.

[96] Gurantz D, Cowling RT, Varki N, Frikovsky E, Moore CD, Greenberg BH (2005). IL-1beta and TNF-alpha upregulate angiotensin II type 1 (AT1) receptors on cardiac fibroblasts and are associated with increased AT1 density in the post-MI heart. J Mol Cell Cardiol.

[97] Gurantz D, Yndestad A, Halvorsen B, Lunde OV, Omens JH, Ueland T, Aukrust P, Moore CD, Kjekshus J, Greenberg BH (2005). Etanercept or intravenous immunoglobulin attenuates expression of genes involved in post-myocardial infarction remodeling. Cardiovasc Res.

[98] Hamid T, Gu Y, Ortines RV, Bhattacharya C, Wang G, Xuan YT, Prabhu SD (2009). Divergent tumor necrosis factor receptor-related remodeling responses in heart failure: role of nuclear factor-kappaB and inflammatory activation. Circulation.

[99] Hanna A, Frangogiannis NG (2019). The role of the TGF-beta superfamily in myocardial infarction. Front Cardiovasc Med.

[100] Hara H, Friedlander RM, Gagliardini V, Ayata C, Fink K, Huang Z, Shimizu-Sasamata M, Yuan J, Moskowitz MA (1997). Inhibition of interleukin 1beta converting enzyme family proteases reduces ischemic and excitotoxic neuronal damage. Proc Natl Acad Sci USA.

[101] Harouki N, Nicol L, Remy-Jouet I, Henry JP, Dumesnil A, Lejeune A, Renet S, Golding F, Djerada Z, Wecker D, Bolduc V, Bouly M, Roussel J, Richard V, Mulder P (2017). The IL-1beta antibody gevokizumab limits cardiac remodeling and coronary dysfunction in rats with heart failure. JACC Basic Transl Sci.

[102] Hartman MH, Vreeswijk-Baudoin I, Groot HE, van de Kolk KW, de Boer RA, Mateo Leach I, Vliegenthart R, Sillje HH, van der Harst P (2016). Inhibition of interleukin-6 receptor in a murine model of myocardial ischemia-reperfusion. PLoS ONE.

[103] Hawrylowicz CM, Santoro SA, Platt FM, Unanue ER (1989). Activated platelets express IL-1 activity. J Immunol.

[104] Hosomi N, Ban CR, Naya T, Takahashi T, Guo P, Song XY, Kohno M (2005). Tumor necrosis factor-alpha neutralization reduced cerebral edema through inhibition of matrix metalloproteinase production after transient focal cerebral ischemia. J Cereb Blood Flow Metab.

[105] Huber SA, Sakkinen P, Conze D, Hardin N, Tracy R (1999). Interleukin-6 exacerbates early atherosclerosis in mice. Arterioscler Thromb Vasc Biol.

[106] Huber SA, Sakkinen P, David C, Newell MK, Tracy RP (2001). T helper-cell phenotype regulates atherosclerosis in mice under conditions of mild hypercholesterolemia. Circulation.

[107] Hwang MW, Matsumori A, Furukawa Y, Ono K, Okada M, Iwasaki A, Hara M, Miyamoto T, Touma M, Sasayama S (2001). Neutralization of interleukin-1beta in the acute phase of myocardial infarction promotes the progression of left ventricular remodeling. J Am Coll Cardiol.

[108] Ibanez B, Aletras AH, Arai AE, Arheden H, Bax J, Berry C, Bucciarelli-Ducci C, Croisille P, Dall'Armellina E, Dharmakumar R, Eitel I, Fernandez-Jimenez R, Friedrich MG, Garcia-Dorado D, Hausenloy DJ, Kim RJ, Kozerke S, Kramer CM, Salerno M, Sanchez-Gonzalez J, Sanz J, Fuster V (2019). Cardiac MRI endpoints in myocardial infarction experimental and clinical trials: JACC scientific expert panel. J Am Coll Cardiol.

[109] Ip WKE, Hoshi N, Shouval DS, Snapper S, Medzhitov R (2017). Anti-inflammatory effect of IL-10 mediated by metabolic reprogramming of macrophages. Science.

[110] Isoda K, Sawada S, Ishigami N, Matsuki T, Miyazaki K, Kusuhara M, Iwakura Y, Ohsuzu F (2004). Lack of interleukin-1 receptor antagonist modulates plaque composition in apolipoprotein E-deficient mice. Arterioscler Thromb Vasc Biol.

[111] Iwata N, Takayama H, Xuan M, Kamiuchi S, Matsuzaki H, Okazaki M, Hibino Y (2015). Effects of etanercept against transient cerebral ischemia in diabetic rats. Biomed Res Int.

[112] Jin JO, Han X, Yu Q (2013). Interleukin-6 induces the generation of IL-10-producing Tr1 cells and suppresses autoimmune tissue inflammation. J Autoimmun.

[113] Jong WM, Ten Cate H, Linnenbank AC, de Boer OJ, Reitsma PH, de Winter RJ, Zuurbier CJ (2016). Reduced acute myocardial ischemia-reperfusion injury in IL-6-deficient mice employing a closed-chest model. Inflamm Res.

[114] Jovinge S, Ares MP, Kallin B, Nilsson J (1996). Human monocytes/macrophages release TNF-alpha in response to Ox-LDL. Arterioscler Thromb Vasc Biol.

[115] Jung M, Ma Y, Iyer RP, DeLeon-Pennell KY, Yabluchanskiy A, Garrett MR, Lindsey ML (2017). IL-10 improves cardiac remodeling after myocardial infarction by stimulating M2 macrophage polarization and fibroblast activation. Basic Res Cardiol.

[116] Kamimura M, Viedt C, Dalpke A, Rosenfeld ME, Mackman N, Cohen DM, Blessing E, Preusch M, Weber CM, Kreuzer J, Katus HA, Bea F (2005). Interleukin-10 suppresses tissue factor expression in lipopolysaccharide-stimulated macrophages via inhibition of Egr-1 and a serum response element/MEK-ERK1/2 pathway. Circ Res.

[117] Kany S, Vollrath JT, Relja B (2019). Cytokines in inflammatory disease. Int J Mol Sci.

[118] King A, Balaji S, Le LD, Crombleholme TM, Keswani SG (2014). Regenerative wound healing: the role of interleukin-10. Adv Wound Care (New Rochelle).

[119] King VL, Szilvassy SJ, Daugherty A (2002). Interleukin-4 deficiency decreases atherosclerotic lesion formation in a site-specific manner in female LDL receptor-/- mice. Arterioscler Thromb Vasc Biol.

[120] Kirii H, Niwa T, Yamada Y, Wada H, Saito K, Iwakura Y, Asano M, Moriwaki H, Seishima M (2003). Lack of interleukin-1beta decreases the severity of atherosclerosis in ApoE-deficient mice. Arterioscler Thromb Vasc Biol.

[121] Kirk B, Feehan J, Lombardi G, Duque G (2020). Muscle, bone, and fat crosstalk: the biological role of myokines, osteokines, and adipokines. Curr Osteoporos Rep.

[122] Kisiel B, Kruszewski R, Juszkiewicz A, Raczkiewicz A, Bachta A, Tlustochowicz M, Staniszewska-Varga J, Klos K, Duda K, Boguslawska-Walecka R, Ploski R, Tlustochowicz W (2015). Methotrexate, cyclosporine A, and biologics protect against atherosclerosis in rheumatoid arthritis. J Immunol Res.

[123] Kleinbongard P, Bose D, Baars T, Mohlenkamp S, Konorza T, Schoner S, Elter-Schulz M, Eggebrecht H, Degen H, Haude M, Levkau B, Schulz R, Erbel R, Heusch G (2011). Vasoconstrictor potential of coronary aspirate from patients undergoing stenting of saphenous vein aortocoronary bypass grafts and its pharmacological attenuation. Circ Res.

[124] Kleinbongard P, Konorza T, Bose D, Baars T, Haude M, Erbel R, Heusch G (2012). Lessons from human coronary aspirate. J Mol Cell Cardiol.

[125] Kleinbongard P, Schulz R, Heusch G (2011). TNFalpha in myocardial ischemia/reperfusion, remodeling and heart failure. Heart Fail Rev.

[126] Kleveland O, Kunszt G, Bratlie M, Ueland T, Broch K, Holte E, Michelsen AE, Bendz B, Amundsen BH, Espevik T, Aakhus S, Damas JK, Aukrust P, Wiseth R, Gullestad L (2016). Effect of a single dose of the interleukin-6 receptor antagonist tocilizumab on inflammation and troponin T release in patients with non-ST-elevation myocardial infarction: a double-blind, randomized, placebo-controlled phase 2 trial. Eur Heart J.

[127] Kober F, Canault M, Peiretti F, Mueller C, Kopp F, Alessi MC, Cozzone PJ, Nalbone G, Bernard M (2007). MRI follow-up of TNF-dependent differential progression of atherosclerotic wall-thickening in mouse aortic arch from early to advanced stages. Atherosclerosis.

[128] Koch C, Engele J (2020). Functions of the CXCL12 receptor ACKR3/CXCR7-what has been perceived and what has been overlooked. Mol Pharmacol.

[129] Krishnamurthy P, Rajasingh J, Lambers E, Qin G, Losordo DW, Kishore R (2009). IL-10 inhibits inflammation and attenuates left ventricular remodeling after myocardial infarction via activation of STAT3 and suppression of HuR. Circ Res.

[130] Kume K, Amano K, Yamada S, Kanazawa T, Ohta H, Hatta K, Amano K, Kuwaba N (2017). Tofacitinib improves atherosclerosis despite up-regulating serum cholesterol in patients with active rheumatoid arthritis: a cohort study. Rheumatol Int.

[131] Labruto F, Yang J, Vaage J, Valen G (2005). Role of tumor necrosis factor alpha and its receptor I in preconditioning by hyperoxia. Basic Res Cardiol.

[132] Lambertsen KL, Clausen BH, Babcock AA, Gregersen R, Fenger C, Nielsen HH, Haugaard LS, Wirenfeldt M, Nielsen M, Dagnaes-Hansen F, Bluethmann H, Faergeman NJ, Meldgaard M, Deierborg T, Finsen B (2009). Microglia protect neurons against ischemia by synthesis of tumor necrosis factor. J Neurosci.

[133] Lawler PR, Bhatt DL, Godoy LC, Luscher TF, Bonow RO, Verma S, Ridker PM (2020). Targeting cardiovascular inflammation: next steps in clinical translation. Eur Heart J.

[134] Lee IT, Lin CC, Wu YC, Yang CM (2010). TNF-alpha induces matrix metalloproteinase-9 expression in A549 cells: role of TNFR1/TRAF2/PKCalpha-dependent signaling pathways. J Cell Physiol.

[135] Lee JH, Kam EH, Kim JM, Kim SY, Kim EJ, Cheon SY, Koo BN (2017). Intranasal administration of interleukin-1 receptor antagonist in a transient focal cerebral ischemia rat model. Biomol Ther (Seoul).

[136] Lessard MR, Trepanier CA, Gourdeau M, Denault PH (1988). A microbiological study of the contamination of the syringes used in anaesthesia practice. Can J Anaesth.

[137] Li D, Zhao L, Liu M, Du X, Ding W, Zhang J, Mehta JL (1999). Kinetics of tumor necrosis factor alpha in plasma and the cardioprotective effect of a monoclonal antibody to tumor necrosis factor alpha in acute myocardial infarction. Am Heart J.

[138] Libby P (2002). Inflammation in atherosclerosis. Nature.

[139] Liberale L, Bonetti NR, Puspitasari YM, Schwarz L, Akhmedov A, Montecucco F, Ruschitzka F, Beer JH, Luscher TF, Simard J, Libby P, Camici GG (2020). Postischemic administration of IL-1alpha neutralizing antibody reduces brain damage and neurological deficit in experimental stroke. Circulation.

[140] Liberale L, Carbone F, Montecucco F, Sahebkar A (2020). Statins reduce vascular inflammation in atherogenesis: a review of underlying molecular mechanisms. Int J Biochem Cell Biol.

[141] Liberale L, Dallegri F, Montecucco F, Carbone F (2017). Pathophysiological relevance of macrophage subsets in atherogenesis. Thromb Haemost.

[142] Liberale L, Diaz-Canestro C, Bonetti NR, Paneni F, Akhmedov A, Beer JH, Montecucco F, Luscher TF, Camici GG (2018). Post-ischaemic administration of the murine Canakinumab-surrogate antibody improves outcome in experimental stroke. Eur Heart J.

[143] Liberale L, Holy EW, Akhmedov A, Bonetti NR, Nietlispach F, Matter CM, Mach F, Montecucco F, Beer JH, Paneni F, Ruschitzka F, Libby P, Luscher TF, Camici GG (2019). Interleukin-1beta mediates arterial thrombus formation via NET-associated tissue factor. J Clin Med.

[144] Liberale L, Montecucco F, Camici GG, Dallegri F, Vecchie A, Carbone F, Bonaventura A (2017). Treatment with proprotein convertase subtilisin/kexin type 9 (PCSK9) inhibitors to reduce cardiovascular inflammation and outcomes. Curr Med Chem.

[145] Liberale L, Montecucco F, Schwarz L, Luscher TF, Camici GG (2020). Inflammation and cardiovascular diseases: lessons from seminal clinical trials. Cardiovasc Res.

[146] Liberale L, Montecucco F, Tardif JC, Libby P, Camici GG (2020). Inflamm-ageing: the role of inflammation in age-dependent cardiovascular disease. Eur Heart J.

[147] Liesz A, Bauer A, Hoheisel JD, Veltkamp R (2014). Intracerebral interleukin-10 injection modulates post-ischemic neuroinflammation: an experimental microarray study. Neurosci Lett.

[148] Liguz-Lecznar M, Zakrzewska R, Kossut M (2015). Inhibition of Tnf-alpha R1 signaling can rescue functional cortical plasticity impaired in early post-stroke period. Neurobiol Aging.

[149] Lim JH, Um HJ, Park JW, Lee IK, Kwon TK (2009). Interleukin-1beta promotes the expression of monocyte chemoattractant protein-1 in human aorta smooth muscle cells via multiple signaling pathways. Exp Mol Med.

[150] Liu X, Liu J, Zhao S, Zhang H, Cai W, Cai M, Ji X, Leak RK, Gao Y, Chen J, Hu X (2016). Interleukin-4 is essential for microglia/macrophage M2 polarization and long-term recovery after cerebral ischemia. Stroke.

[151] Loddick SA, Rothwell NJ (1996). Neuroprotective effects of human recombinant interleukin-1 receptor antagonist in focal cerebral ischaemia in the rat. J Cereb Blood Flow Metab.

[152] Loddick SA, Wong ML, Bongiorno PB, Gold PW, Licinio J, Rothwell NJ (1997). Endogenous interleukin-1 receptor antagonist is neuroprotective. Biochem Biophys Res Commun.

[153] Madsen PM, Clausen BH, Degn M, Thyssen S, Kristensen LK, Svensson M, Ditzel N, Finsen B, Deierborg T, Brambilla R, Lambertsen KL (2016). Genetic ablation of soluble tumor necrosis factor with preservation of membrane tumor necrosis factor is associated with neuroprotection after focal cerebral ischemia. J Cereb Blood Flow Metab.

[154] Maekawa N, Wada H, Kanda T, Niwa T, Yamada Y, Saito K, Fujiwara H, Sekikawa K, Seishima M (2002). Improved myocardial ischemia/reperfusion injury in mice lacking tumor necrosis factor-alpha. J Am Coll Cardiol.

[155] Mallat Z, Besnard S, Duriez M, Deleuze V, Emmanuel F, Bureau MF, Soubrier F, Esposito B, Duez H, Fievet C, Staels B, Duverger N, Scherman D, Tedgui A (1999). Protective role of interleukin-10 in atherosclerosis. Circ Res.

[156] Mallat Z, Heymes C, Ohan J, Faggin E, Leseche G, Tedgui A (1999). Expression of interleukin-10 in advanced human atherosclerotic plaques: relation to inducible nitric oxide synthase expression and cell death. Arterioscler Thromb Vasc Biol.

[157] Matsushita K, Iwanaga S, Oda T, Kimura K, Shimada M, Sano M, Umezawa A, Hata J, Ogawa S (2005). Interleukin-6/soluble interleukin-6 receptor complex reduces infarct size via inhibiting myocardial apoptosis. Lab Invest.

[158] Mauer J, Denson JL, Bruning JC (2015). Versatile functions for IL-6 in metabolism and cancer. Trends Immunol.

[159] Mauro AG, Mezzaroma E, Torrado J, Kundur P, Joshi P, Stroud K, Quaini F, Lagrasta CA, Abbate A, Toldo S (2017). Reduction of myocardial ischemia-reperfusion injury by inhibiting interleukin-1 alpha. J Cardiovasc Pharmacol.

[160] Maysami S, Wong R, Pradillo JM, Denes A, Dhungana H, Malm T, Koistinaho J, Orset C, Rahman M, Rubio M, Schwaninger M, Vivien D, Bath PM, Rothwell NJ, Allan SM (2016). A cross-laboratory preclinical study on the effectiveness of interleukin-1 receptor antagonist in stroke. J Cereb Blood Flow Metab.

[161] McAlpine CS, Kiss MG, Rattik S, He S, Vassalli A, Valet C, Anzai A, Chan CT, Mindur JE, Kahles F, Poller WC, Frodermann V, Fenn AM, Gregory AF, Halle L, Iwamoto Y, Hoyer FF, Binder CJ, Libby P, Tafti M, Scammell TE, Nahrendorf M, Swirski FK (2019). Sleep modulates haematopoiesis and protects against atherosclerosis. Nature.

[162] McCulloch L, Allan SM, Emsley HC, Smith CJ, McColl BW (2019). Interleukin-1 receptor antagonist treatment in acute ischaemic stroke does not alter systemic markers of anti-microbial defence. F1000Res.

[163] Merhi-Soussi F, Kwak BR, Magne D, Chadjichristos C, Berti M, Pelli G, James RW, Mach F, Gabay C (2005). Interleukin-1 plays a major role in vascular inflammation and atherosclerosis in male apolipoprotein E-knockout mice. Cardiovasc Res.

[164] Moe GW, Marin-Garcia J, Konig A, Goldenthal M, Lu X, Feng Q (2004). In vivo TNF-alpha inhibition ameliorates cardiac mitochondrial dysfunction, oxidative stress, and apoptosis in experimental heart failure. Am J Physiol Heart Circ Physiol.

[165] Mog B, Asase C, Chaplin A, Gao H, Rajagopalan S, Maiseyeu A (2019). Nano-antagonist alleviates inflammation and allows for MRI of atherosclerosis. Nanotheranostics.

[166] Mohr T, Haudek-Prinz V, Slany A, Grillari J, Micksche M, Gerner C (2017). Proteome profiling in IL-1beta and VEGF-activated human umbilical vein endothelial cells delineates the interlink between inflammation and angiogenesis. PLoS ONE.

[167] Monden Y, Kubota T, Inoue T, Tsutsumi T, Kawano S, Ide T, Tsutsui H, Sunagawa K (2007). Tumor necrosis factor-alpha is toxic via receptor 1 and protective via receptor 2 in a murine model of myocardial infarction. Am J Physiol Heart Circ Physiol.

[168] Morton AC, Arnold ND, Gunn J, Varcoe R, Francis SE, Dower SK, Crossman DC (2005). Interleukin-1 receptor antagonist alters the response to vessel wall injury in a porcine coronary artery model. Cardiovasc Res.

[169] Nash M, McGrath JP, Cartland SP, Patel S, Kavurma MM (2019). Tumour necrosis factor superfamily members in ischaemic vascular diseases. Cardiovasc Res.

[170] Nawashiro H, Martin D, Hallenbeck JM (1997). Inhibition of tumor necrosis factor and amelioration of brain infarction in mice. J Cereb Blood Flow Metab.

[171] Nawashiro H, Martin D, Hallenbeck JM (1997). Neuroprotective effects of TNF binding protein in focal cerebral ischemia. Brain Res.

[172] Newman WH, Castresana MR, Webb JG, Wang Z (2003). Cyclic AMP inhibits production of interleukin-6 and migration in human vascular smooth muscle cells. J Surg Res.

[173] Noels H, Weber C, Koenen RR (2019). Chemokines as therapeutic targets in cardiovascular disease. Arterioscler Thromb Vasc Biol.

[174] Ohta H, Wada H, Niwa T, Kirii H, Iwamoto N, Fujii H, Saito K, Sekikawa K, Seishima M (2005). Disruption of tumor necrosis factor-alpha gene diminishes the development of atherosclerosis in ApoE-deficient mice. Atherosclerosis.

[175] Ooboshi H, Ibayashi S, Shichita T, Kumai Y, Takada J, Ago T, Arakawa S, Sugimori H, Kamouchi M, Kitazono T, Iida M (2005). Postischemic gene transfer of interleukin-10 protects against both focal and global brain ischemia. Circulation.

[176] Ottonello L, Morone MP, Dapino P, Dallegri F (1995). Tumour necrosis factor alpha-induced oxidative burst in neutrophils adherent to fibronectin: effects of cyclic AMP-elevating agents. Br J Haematol.

[177] Ozen G, Pedro S, Michaud K (2020). The risk of cardiovascular events associated with disease-modifying antirheumatic drugs in rheumatoid arthritis. J Rheumatol.

[178] Padfield GJ, Din JN, Koushiappi E, Mills NL, Robinson SD, Cruden Nle M, Lucking AJ, Chia S, Harding SA, Newby DE (2013). Cardiovascular effects of tumour necrosis factor alpha antagonism in patients with acute myocardial infarction: a first in human study. Heart.

[179] Perez-de Puig I, Miro F, Salas-Perdomo A, Bonfill-Teixidor E, Ferrer-Ferrer M, Marquez-Kisinousky L, Planas AM (2013). IL-10 deficiency exacerbates the brain inflammatory response to permanent ischemia without preventing resolution of the lesion. J Cereb Blood Flow Metab.

[180] Picchi A, Gao X, Belmadani S, Potter BJ, Focardi M, Chilian WM, Zhang C (2006). Tumor necrosis factor-alpha induces endothelial dysfunction in the prediabetic metabolic syndrome. Circ Res.

[181] Pickering M, Cumiskey D, O'Connor JJ (2005). Actions of TNF-alpha on glutamatergic synaptic transmission in the central nervous system. Exp Physiol.

[182] Pignatelli P, De Biase L, Lenti L, Tocci G, Brunelli A, Cangemi R, Riondino S, Grego S, Volpe M, Violi F (2005). Tumor necrosis factor-alpha as trigger of platelet activation in patients with heart failure. Blood.

[183] Pinderski LJ, Fischbein MP, Subbanagounder G, Fishbein MC, Kubo N, Cheroutre H, Curtiss LK, Berliner JA, Boisvert WA (2002). Overexpression of interleukin-10 by activated T lymphocytes inhibits atherosclerosis in LDL receptor-deficient Mice by altering lymphocyte and macrophage phenotypes. Circ Res.

[184] Pircher J, Merkle M, Wornle M, Ribeiro A, Czermak T, Stampnik Y, Mannell H, Niemeyer M, Vielhauer V, Krotz F (2012). Prothrombotic effects of tumor necrosis factor alpha in vivo are amplified by the absence of TNF-alpha receptor subtype 1 and require TNF-alpha receptor subtype 2. Arthritis Res Ther.

[185] Polunovsky VA, Wendt CH, Ingbar DH, Peterson MS, Bitterman PB (1994). Induction of endothelial cell apoptosis by TNF alpha: modulation by inhibitors of protein synthesis. Exp Cell Res.

[186] Potteaux S, Esposito B, van Oostrom O, Brun V, Ardouin P, Groux H, Tedgui A, Mallat Z (2004). Leukocyte-derived interleukin 10 is required for protection against atherosclerosis in low-density lipoprotein receptor knockout mice. Arterioscler Thromb Vasc Biol.

[187] Qin M, Wang W, Zhou H, Wang X, Wang F, Wang H (2020). Circular RNA circ_0003645 silencing alleviates inflammation and apoptosis via the NF-kappaB pathway in endothelial cells induced by oxLDL. Gene.

[188] Rajamaki K, Lappalainen J, Oorni K, Valimaki E, Matikainen S, Kovanen PT, Eklund KK (2010). Cholesterol crystals activate the NLRP3 inflammasome in human macrophages: a novel link between cholesterol metabolism and inflammation. PLoS ONE.

[189] Ramani R, Mathier M, Wang P, Gibson G, Togel S, Dawson J, Bauer A, Alber S, Watkins SC, McTiernan CF, Feldman AM (2004). Inhibition of tumor necrosis factor receptor-1-mediated pathways has beneficial effects in a murine model of postischemic remodeling. Am J Physiol Heart Circ Physiol.

[190] Rastogi S, Rizwani W, Joshi B, Kunigal S, Chellappan SP (2012). TNF-alpha response of vascular endothelial and vascular smooth muscle cells involve differential utilization of ASK1 kinase and p73. Cell Death Differ.

[191] Relton JK, Martin D, Thompson RC, Russell DA (1996). Peripheral administration of Interleukin-1 Receptor antagonist inhibits brain damage after focal cerebral ischemia in the rat. Exp Neurol.

[192] Ridker PM, Everett BM, Thuren T, MacFadyen JG, Chang WH, Ballantyne C, Fonseca F, Nicolau J, Koenig W, Anker SD, Kastelein JJP, Cornel JH, Pais P, Pella D, Genest J, Cifkova R, Lorenzatti A, Forster T, Kobalava Z, Vida-Simiti L, Flather M, Shimokawa H, Ogawa H, Dellborg M, Rossi PRF, Troquay RPT, Libby P, Glynn RJ, Group CT (2017). Antiinflammatory therapy with canakinumab for atherosclerotic disease. N Engl J Med.

[193] Rizzo FR, Musella A, De Vito F, Fresegna D, Bullitta S, Vanni V, Guadalupi L, Stampanoni Bassi M, Buttari F, Mandolesi G, Centonze D, Gentile A (2018). Tumor necrosis factor and interleukin-1beta modulate synaptic plasticity during neuroinflammation. Neural Plast.

[194] Robert R, Chapelain B, Jean T, Neliat G (1992). Interleukin-1 impairs both vascular contraction and relaxation in rabbit isolated aorta. Biochem Biophys Res Commun.

[195] Ross R, Glomset JA (1976). The pathogenesis of atherosclerosis (first of two parts). N Engl J Med.

[196] Roubille C, Richer V, Starnino T, McCourt C, McFarlane A, Fleming P, Siu S, Kraft J, Lynde C, Pope J, Gulliver W, Keeling S, Dutz J, Bessette L, Bissonnette R, Haraoui B (2015). The effects of tumour necrosis factor inhibitors, methotrexate, non-steroidal anti-inflammatory drugs and corticosteroids on cardiovascular events in rheumatoid arthritis, psoriasis and psoriatic arthritis: a systematic review and meta-analysis. Ann Rheum Dis.

[197] Ruiz-Limon P, Ortega R, Arias de la Rosa I, Abalos-Aguilera MDC, Perez-Sanchez C, Jimenez-Gomez Y, Peralbo-Santaella E, Font P, Ruiz-Vilches D, Ferrin G, Collantes-Estevez E, Escudero-Contreras A, Lopez-Pedrera C, Barbarroja N (2017). Tocilizumab improves the proatherothrombotic profile of rheumatoid arthritis patients modulating endothelial dysfunction, NETosis, and inflammation. Transl Res.

[198] Rus HG, Niculescu F, Vlaicu R (1991). Tumor necrosis factor-alpha in human arterial wall with atherosclerosis. Atherosclerosis.

[199] Salmeron K, Aihara T, Redondo-Castro E, Pinteaux E, Bix G (2016). IL-1alpha induces angiogenesis in brain endothelial cells in vitro: implications for brain angiogenesis after acute injury. J Neurochem.

[200] Salmeron KE, Maniskas ME, Edwards DN, Wong R, Rajkovic I, Trout A, Rahman AA, Hamilton S, Fraser JF, Pinteaux E, Bix GJ (2019). Interleukin 1 alpha administration is neuroprotective and neuro-restorative following experimental ischemic stroke. J Neuroinflamm.

[201] Sasu S, Beasley D (2000). Essential roles of IkappaB kinases alpha and beta in serum- and IL-1-induced human VSMC proliferation. Am J Physiol Heart Circ Physiol.

[202] Savard A, Brochu ME, Chevin M, Guiraut C, Grbic D, Sebire G (2015). Neuronal self-injury mediated by IL-1beta and MMP-9 in a cerebral palsy model of severe neonatal encephalopathy induced by immune activation plus hypoxia-ischemia. J Neuroinflamm.

[203] Scheller J, Chalaris A, Schmidt-Arras D, Rose-John S (2011). The pro- and anti-inflammatory properties of the cytokine interleukin-6. Biochim Biophys Acta.

[204] Schieffer B, Selle T, Hilfiker A, Hilfiker-Kleiner D, Grote K, Tietge UJ, Trautwein C, Luchtefeld M, Schmittkamp C, Heeneman S, Daemen MJ, Drexler H (2004). Impact of interleukin-6 on plaque development and morphology in experimental atherosclerosis. Circulation.

[205] Schmidt-Arras D, Rose-John S (2016). IL-6 pathway in the liver: from physiopathology to therapy. J Hepatol.

[206] Schonbeck U, Mach F, Bonnefoy JY, Loppnow H, Flad HD, Libby P (1997). Ligation of CD40 activates interleukin 1beta-converting enzyme (caspase-1) activity in vascular smooth muscle and endothelial cells and promotes elaboration of active interleukin 1beta. J Biol Chem.

[207] Schreyer SA, Peschon JJ, LeBoeuf RC (1996). Accelerated atherosclerosis in mice lacking tumor necrosis factor receptor p55. J Biol Chem.

[208] Schultz K, Murthy V, Tatro JB, Beasley D (2007). Endogenous interleukin-1 alpha promotes a proliferative and proinflammatory phenotype in human vascular smooth muscle cells. Am J Physiol Heart Circ Physiol.

[209] Schulz R, Heusch G (2009). Tumor necrosis factor-alpha and its receptors 1 and 2: Yin and Yang in myocardial infarction?. Circulation.

[210] Schumacher SM, Naga Prasad SV (2018). Tumor necrosis factor-alpha in heart failure: an updated review. Curr Cardiol Rep.

[211] Shimokawa H, Ito A, Fukumoto Y, Kadokami T, Nakaike R, Sakata M, Takayanagi T, Egashira K, Takeshita A (1996). Chronic treatment with interleukin-1 beta induces coronary intimal lesions and vasospastic responses in pigs in vivo. The role of platelet-derived growth factor. J Clin Invest.

[212] Skyschally A, Gres P, Hoffmann S, Haude M, Erbel R, Schulz R, Heusch G (2007). Bidirectional role of tumor necrosis factor-alpha in coronary microembolization: progressive contractile dysfunction versus delayed protection against infarction. Circ Res.

[213] Smith CJ, Hulme S, Vail A, Heal C, Parry-Jones AR, Scarth S, Hopkins K, Hoadley M, Allan SM, Rothwell NJ, Hopkins SJ, Tyrrell PJ (2018). SCIL-STROKE (subcutaneous interleukin-1 receptor antagonist in ischemic stroke): a randomized controlled phase 2 trial. Stroke.

[214] Smith E, Prasad KM, Butcher M, Dobrian A, Kolls JK, Ley K, Galkina E (2010). Blockade of interleukin-17A results in reduced atherosclerosis in apolipoprotein E-deficient mice. Circulation.

[215] Sobowale OA, Parry-Jones AR, Smith CJ, Tyrrell PJ, Rothwell NJ, Allan SM (2016). Interleukin-1 in stroke: from bench to bedside. Stroke.

[216] Spera PA, Ellison JA, Feuerstein GZ, Barone FC (1998). IL-10 reduces rat brain injury following focal stroke. Neurosci Lett.

[217] Spulber S, Bartfai T, Schultzberg M (2009). IL-1/IL-1ra balance in the brain revisited—evidence from transgenic mouse models. Brain Behav Immun.

[218] Sugano M, Tsuchida K, Hata T, Makino N (2004). In vivo transfer of soluble TNF-alpha receptor 1 gene improves cardiac function and reduces infarct size after myocardial infarction in rats. FASEB J.

[219] Sumbria RK, Boado RJ, Pardridge WM (2012). Brain protection from stroke with intravenous TNFalpha decoy receptor-Trojan horse fusion protein. J Cereb Blood Flow Metab.

[220] Sun HJ, Ren XS, Xiong XQ, Chen YZ, Zhao MX, Wang JJ, Zhou YB, Han Y, Chen Q, Li YH, Kang YM, Zhu GQ (2017). NLRP3 inflammasome activation contributes to VSMC phenotypic transformation and proliferation in hypertension. Cell Death Dis.

[221] Suzuki K, Murtuza B, Smolenski RT, Sammut IA, Suzuki N, Kaneda Y, Yacoub MH (2001). Overexpression of interleukin-1 receptor antagonist provides cardioprotection against ischemia-reperfusion injury associated with reduction in apoptosis. Circulation.

[222] Swiatkowska M, Szemraj J, Cierniewski CS (2005). Induction of PAI-1 expression by tumor necrosis factor alpha in endothelial cells is mediated by its responsive element located in the 4G/5G site. FEBS J.

[223] Teng X, Zhang H, Snead C, Catravas JD (2002). Molecular mechanisms of iNOS induction by IL-1 beta and IFN-gamma in rat aortic smooth muscle cells. Am J Physiol Cell Physiol.

[224] Thompson WL, Van Eldik LJ (2009). Inflammatory cytokines stimulate the chemokines CCL2/MCP-1 and CCL7/MCP-3 through NFkB and MAPK dependent pathways in rat astrocytes [corrected]. Brain Res.

[225] Tilg H, Trehu E, Atkins MB, Dinarello CA, Mier JW (1994). Interleukin-6 (IL-6) as an anti-inflammatory cytokine: induction of circulating IL-1 receptor antagonist and soluble tumor necrosis factor receptor p55. Blood.

[226] Toldo S, Mauro AG, Cutter Z, Van Tassell BW, Mezzaroma E, Del Buono MG, Prestamburgo A, Potere N, Abbate A (2019). The NLRP3 inflammasome inhibitor, OLT1177 (dapansutrile), reduces infarct size and preserves contractile function after ischemia reperfusion injury in the mouse. J Cardiovasc Pharmacol.

[227] Toldo S, Mezzaroma E, Bressi E, Marchetti C, Carbone S, Sonnino C, Van Tassell BW, Abbate A (2014). Interleukin-1beta blockade improves left ventricular systolic/diastolic function and restores contractility reserve in severe ischemic cardiomyopathy in the mouse. J Cardiovasc Pharmacol.

[228] Toldo S, Mezzaroma E, Van Tassell BW, Farkas D, Marchetti C, Voelkel NF, Abbate A (2013). Interleukin-1beta blockade improves cardiac remodelling after myocardial infarction without interrupting the inflammasome in the mouse. Exp Physiol.

[229] Toldo S, Schatz AM, Mezzaroma E, Chawla R, Stallard TW, Stallard WC, Jahangiri A, Van Tassell BW, Abbate A (2012). Recombinant human interleukin-1 receptor antagonist provides cardioprotection during myocardial ischemia reperfusion in the mouse. Cardiovasc Drugs Ther.

[230] Tosato G, Jones KD (1990). Interleukin-1 induces interleukin-6 production in peripheral blood monocytes. Blood.

[231] Toufektsian MC, Robbez-Masson V, Sanou D, Jouan MG, Ormezzano O, de Leiris J, Boucher F (2008). A single intravenous sTNFR-Fc administration at the time of reperfusion limits infarct size–implications in reperfusion strategies in man. Cardiovasc Drugs Ther.

[232] Tourneur L, Chiocchia G (2010). FADD: a regulator of life and death. Trends Immunol.

[233] Touzani O, Boutin H, LeFeuvre R, Parker L, Miller A, Luheshi G, Rothwell N (2002). Interleukin-1 influences ischemic brain damage in the mouse independently of the interleukin-1 type I receptor. J Neurosci.

[234] Trankle CR, Canada JM, Cei L, Abouzaki N, Oddi-Erdle C, Kadariya D, Christopher S, Viscusi M, Del Buono M, Kontos MC, Arena R, Van Tassell B, Abbate A (2018). Usefulness of canakinumab to improve exercise capacity in patients with long-term systolic heart failure and elevated C-reactive protein. Am J Cardiol.

[235] van Es T, van Puijvelde GH, Ramos OH, Segers FM, Joosten LA, van den Berg WB, Michon IM, de Vos P, van Berkel TJ, Kuiper J (2009). Attenuated atherosclerosis upon IL-17R signaling disruption in LDLr deficient mice. Biochem Biophys Res Commun.

[236] Van Tassell BW, Varma A, Salloum FN, Das A, Seropian IM, Toldo S, Smithson L, Hoke NN, Chau VQ, Robati R, Abbate A (2010). Interleukin-1 trap attenuates cardiac remodeling after experimental acute myocardial infarction in mice. J Cardiovasc Pharmacol.

[237] Venugopal J, Wang J, Mawri J, Guo C, Eitzman D (2020). Interleukin-1 receptor inhibition reduces stroke size in a murine model of sickle cell disease. Haematologica.

[238] Viviani B, Bartesaghi S, Gardoni F, Vezzani A, Behrens MM, Bartfai T, Binaglia M, Corsini E, Di Luca M, Galli CL, Marinovich M (2003). Interleukin-1beta enhances NMDA receptor-mediated intracellular calcium increase through activation of the Src family of kinases. J Neurosci.

[239] Vromman A, Ruvkun V, Shvartz E, Wojtkiewicz G, Santos Masson G, Tesmenitsky Y, Folco E, Gram H, Nahrendorf M, Swirski FK, Sukhova GK, Libby P (2019). Stage-dependent differential effects of interleukin-1 isoforms on experimental atherosclerosis. Eur Heart J.

[240] Wang P, Wu P, Siegel MI, Egan RW, Billah MM (1995). Interleukin (IL)-10 inhibits nuclear factor kappa B (NF kappa B) activation in human monocytes. IL-10 and IL-4 suppress cytokine synthesis by different mechanisms. J Biol Chem.

[241] Wang X, Feuerstein GZ, Gu JL, Lysko PG, Yue TL (1995). Interleukin-1 beta induces expression of adhesion molecules in human vascular smooth muscle cells and enhances adhesion of leukocytes to smooth muscle cells. Atherosclerosis.

[242] Wang X, Guo Z, Ding Z, Mehta JL (2018). Inflammation, autophagy, and apoptosis after myocardial infarction. J Am Heart Assoc.

[243] Williams JW, Huang LH, Randolph GJ (2019). Cytokine circuits in cardiovascular disease. Immunity.

[244] Williams L, Bradley L, Smith A, Foxwell B (2004). Signal transducer and activator of transcription 3 is the dominant mediator of the anti-inflammatory effects of IL-10 in human macrophages. J Immunol.

[245] Works MG, Koenig JB, Sapolsky RM (2013). Soluble TNF receptor 1-secreting ex vivo-derived dendritic cells reduce injury after stroke. J Cereb Blood Flow Metab.

[246] Xiao N, Yin M, Zhang L, Qu X, Du H, Sun X, Mao L, Ren G, Zhang C, Geng Y, An L, Pan J (2009). Tumor necrosis factor-alpha deficiency retards early fatty-streak lesion by influencing the expression of inflammatory factors in apoE-null mice. Mol Genet Metab.

[247] Yamashita T, Sawamoto K, Suzuki S, Suzuki N, Adachi K, Kawase T, Mihara M, Ohsugi Y, Abe K, Okano H (2005). Blockade of interleukin-6 signaling aggravates ischemic cerebral damage in mice: possible involvement of Stat3 activation in the protection of neurons. J Neurochem.

[248] Yan G, You B, Chen SP, Liao JK, Sun J (2008). Tumor necrosis factor-alpha downregulates endothelial nitric oxide synthase mRNA stability via translation elongation factor 1-alpha 1. Circ Res.

[249] Yang Z, Zingarelli B, Szabo C (2000). Crucial role of endogenous interleukin-10 production in myocardial ischemia/reperfusion injury. Circulation.

[250] Yu X, Patterson E, Huang S, Garrett MW, Kem DC (2005). Tumor necrosis factor alpha, rapid ventricular tachyarrhythmias, and infarct size in canine models of myocardial infarction. J Cardiovasc Pharmacol.

[251] Zhang H, Zhang J, Ungvari Z, Zhang C (2009). Resveratrol improves endothelial function: role of TNF{alpha} and vascular oxidative stress. Arterioscler Thromb Vasc Biol.

[252] Zhao TX, Newland SA, Mallat Z (2020). 2019 ATVB plenary lecture: interleukin-2 therapy in cardiovascular disease: the potential to regulate innate and adaptive immunity. Arterioscler Thromb Vasc Biol.

[253] Zhao X, Wang H, Sun G, Zhang J, Edwards NJ, Aronowski J (2015). Neuronal interleukin-4 as a modulator of microglial pathways and ischemic brain damage. J Neurosci.

[254] Zhu J, Huang J, Dai D, Wang X, Gao J, Han W, Zhang R (2019). Recombinant human interleukin-1 receptor antagonist treatment protects rats from myocardial ischemia-reperfusion injury. Biomed Pharmacother.

